# Doublesex regulates *fruitless* expression to promote sexual dimorphism of the gonad stem cell niche

**DOI:** 10.1371/journal.pgen.1009468

**Published:** 2021-03-31

**Authors:** Hong Zhou, Cale Whitworth, Caitlin Pozmanter, Megan C. Neville, Mark Van Doren

**Affiliations:** 1 Department of Biology, Johns Hopkins University, 3400 N. Charles Street, Baltimore, MD, United States of America; 2 Centre for Neural Circuits and Behaviour, University of Oxford, Tinsley Building, Mansfield Road, Oxford, United Kingdom; University of California Davis, UNITED STATES

## Abstract

Doublesex (Dsx) and Fruitless (Fru) are the two downstream transcription factors that actuate *Drosophila* sex determination. While Dsx assists Fru to regulate sex-specific behavior, whether Fru collaborates with Dsx in regulating other aspects of sexual dimorphism remains unknown. One important aspect of sexual dimorphism is found in the gonad stem cell (GSC) niches, where male and female GSCs are regulated to create large numbers of sperm and eggs. Here we report that Fru is expressed male-specifically in the GSC niche and plays important roles in the development and maintenance of these cells. Unlike previously-studied aspects of sex-specific Fru expression, which are regulated by Transformer (Tra)-mediated alternative splicing, we show that male-specific expression of *fru* in the gonad is regulated downstream of *dsx*, and is independent of *tra*. *fru* genetically interacts with *dsx* to support maintenance of the niche throughout development. Ectopic expression of *fru* inhibited female niche formation and partially masculinized the ovary. *fru* is also required autonomously for cyst stem cell maintenance and cyst cell survival. Finally, we identified a conserved Dsx binding site upstream of *fru* promoter *P4* that regulates *fru* expression in the niche, indicating that *fru* is likely a direct target for transcriptional regulation by Dsx. These findings demonstrate that *fru* acts outside the nervous system to influence sexual dimorphism and reveal a new mechanism for regulating sex-specific expression of *fru* that is regulated at the transcriptional level by Dsx, rather than by alternative splicing by Tra.

## Author summary

In animals, the process of sex determination controls the development of sexual dimorphism—the differences in appearance, physiology and behavior observed between males and females of a species. These differences are important for key functions such as sexual reproduction, and also influence other characteristics such as sex-specific disease progression. An important family of transcription factors, the Doublesex, mab-3 Related Transcription factors (DMRTs) control sex-specific development, particularly in the gonads, in most or all animals where they have been studied. Thus, an essential question in biology is how do the DMRTs control sex-specific development? In Drosophila, another set of transcription factors, encoded by the *fruitless (fru)* gene, controls sex-specific development of the nervous system and is thought to be regulated independently of the Drosophila DMRT Doublesex (Dsx). Here we present two important changes to our thinking about sexual development: 1) *fru* also acts to control sex-specific development outside the nervous system (in the gonad) and 2) sex-specific *fru* expression can be regulated by Dsx, in addition to its Dsx-independent regulation, providing a new mechanism for *fru* regulation that may be broadly utilized.

## Introduction

In sexually reproducing animals, the proper production of gametes and successful copulation are equally critical for reproductive success. It is therefore important that both the gonad and the brain know their sexual identity. The Doublesex/Mab-3 Related Transcription Factors (DMRTs) act downstream of sex determination and play an evolutionarily conserved role to establish and maintain sexual dimorphism in the gonad [1]. Meanwhile, sexual dimorphism in other tissues such as the brain is controlled, to varying degrees in different animals, through autonomous control by the sex determination and non-autonomous signaling from the gonads [2,3]. In many invertebrate species, another sex-determination gene *fruitless* (*fru*), which encodes multiple BTB-Zinc finger transcription factors, plays a central role in controlling mate choice, courtship behavior and aggression [4]. How sex determination in the gonad and the nervous system are related and coordinated in these species remains unclear.

The founding member of the DMRT family is *Drosophila doublesex* (*dsx*). *dsx* and *fru* undergo sex-specific alternative mRNA splicing by the sex determination factor Transformer (Tra), together with its co-factor Transformer-2 (Tra-2), to produce transcripts encoding sex-specific protein isoforms. It was once thought that *dsx* controls sexual dimorphism outside the nervous system while *fru* regulates sex-specific nervous system development and behavior. But more recent evidence shows that *dsx* cooperates with *fru* to specify sex-specific neural circuitry and regulate courtship behaviors [5–10]. However, whether *fru* acts along with *dsx* to control sexual dimorphism outside the nervous system remains unknown.

**Fig 1:**
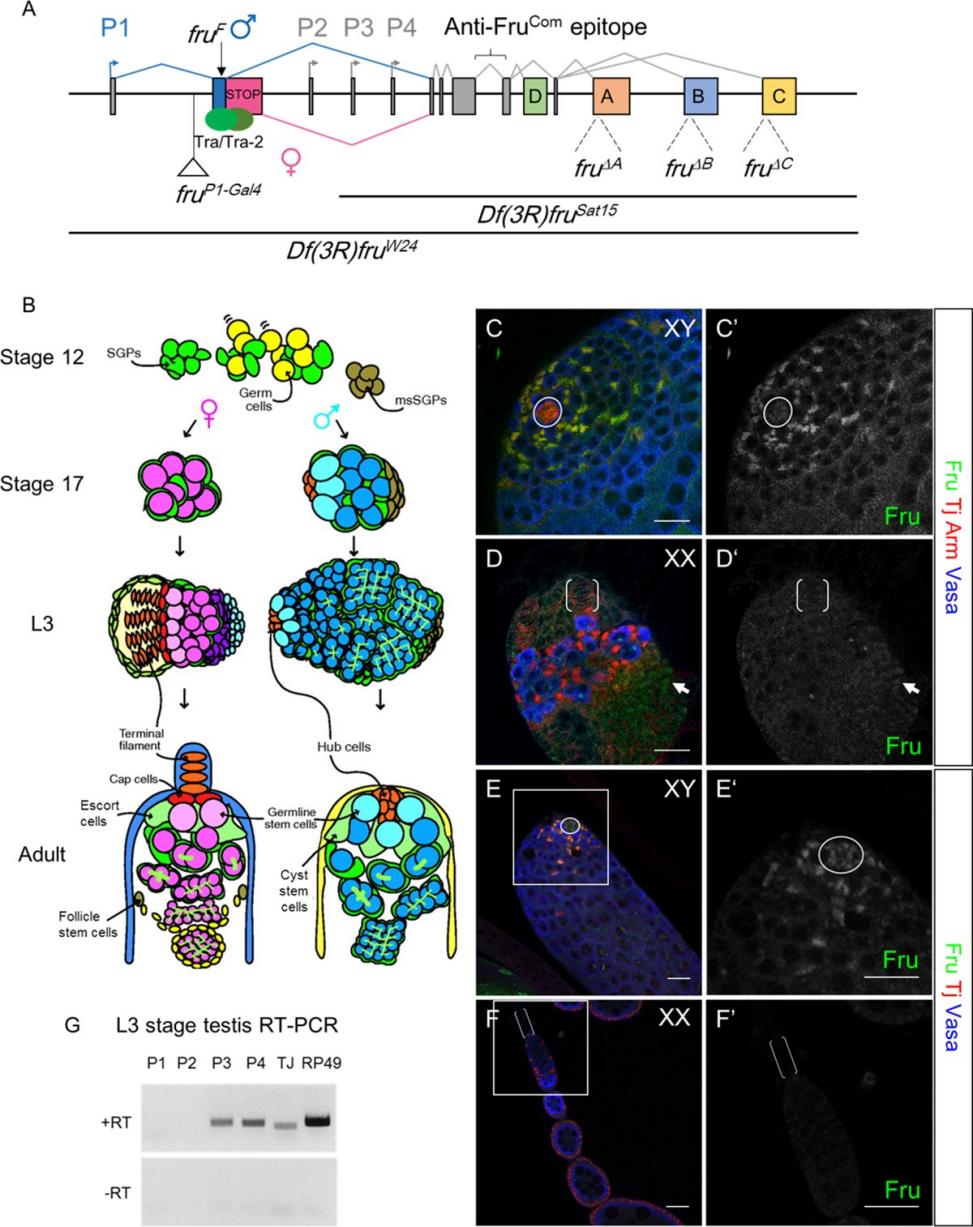
Fruitless is expressed male-specifically in the germline stem cell niche and is independent of Fru^M^. (A) Schematic of the *fruitless* (*fru*) gene locus and alleles utilized in the study. (B) Development of the female and male germline stem cell (GSC) niche. Germ cells are indicated in yellow and then in shades of pink or blue in females and males respectively, with lighter shades indicating the GSCs. Somatic cell types are as labeled. (C-D) Anti-FRU staining in late L3 stage larval gonads. Note that FRU immunoreactivity overlaps with that of the transcription factor TJ. C Anti-vasa (blue) labels the germline, anti-ARM (red) labels the tight cluster of hub cells in males, anti-TJ (also red) labels CySC and early cyst cells in the testis along with somatic cells intermingled with germ cells in the ovary. The arrow in (D) indicates weak Fru expression in terminal epithelial cells. (E-F) Adult testis and ovary. Fru expression in the GSC niche is shown at a higher magnitude in (E’ and F’). Anti-vasa labels the germline and anti-Tj labels the CySC and early cyst cells of the testis along with somatic cells of the ovary. Scale bars represent 20 μm. Circles: hubs; brackets: TFs. (G) RT-PCR of late L3 stage testes with promoter-specific primers. *tj* and *rp49* primers were used as positive controls.

The *fru* gene locus contains a complex transcription unit with multiple promoters and alternative splice forms (Fig 1A). Sex-specific regulation of *fru* was only known to occur through alternative splicing of transcripts produced from the *P1* promoter, which produces the FruM isoforms [11,12]. The downstream promoters (*P2-P4*) produce Fru isoforms (collectively named Fru^Com^) encoded by transcripts that are common to both sexes and are required for viability in both males and females. *fru P1* transcripts have only been detected in the nervous system, suggesting that sex-specific functions of *fru* are limited to neural tissue [13]. However, Fru^Com^ is expressed in several non-neural tissues, including sex-specific cell types of the reproductive system [13,14]. Further, from a recent genome-wide search for putative Dsx targets, we identified *fru* as a candidate for transcriptional regulation by Dsx ([15] and S4 Fig). These data raise the possibility that *fru* functions cooperatively with *dsx* to regulate gonad development.

The stem cell niche is a key component of the gonad that provides signals to regulate the germline stem cells (GSCs) necessary for gametogenesis. Sexual differences within the adult GSC niches have been well-characterized [16]. Important components of the niche are hub cells in males and terminal filaments (TFs) and cap cells in females (Fig 1B). Other important cell types include the cyst stem cells (CySCs) and cyst cells in males and the escort cells, follicle stem cells (FSCs) and follicle cells in females. The hub is a tight cluster of postmitotic cells that forms during the last stages of embryogenesis [17]. In contrast, female niche specification starts in late 3^rd^ larval instar when stacks of terminal filament cells are specified from cells forming the apical cap of the ovary, and continues at the larval-pupal transition with the specification of cap cells from intermingle cells [18–20]. Recently, we found that one important role *dsx* plays is to maintain the hub fate in the 3^rd^ instar larval (L3) stage and to prevent sex reversal [21]. In the absence of *dsx*, both XX and XY gonads initially follow the male path to form a hub by the end of embryogenesis, but later undergo stochastic sexual-fate reprogramming in the L3 stage in which half of both XX and XY animals form TFs in place of the hub, while the hub is maintained in the other half. The genes and pathways that function downstream of *dsx* to regulate male vs. female gonad niche fate remain elusive.

To test if *dsx* and *fru* act in concert to regulate sexual development of the gonad, we investigated *fru* expression and function in the gonad. We found that Fru is expressed male-specifically in the GSC niche and functions to regulate the development and maintenance of the male GSC niche. Sex-specific expression of *fru* is regulated by *dsx*, rather than alternative splicing by Tra. Our analyses show that *fru* is required in *dsx* mutant gonads to prevent hub-to-TF fate conversion and is sufficient to partially masculinize the developing female GSC niche. *fru* also functions in the cyst stem cell (CySC) lineage to maintain CySC fate. Finally, we show that *fru P4* promoter is directly regulated by Dsx, through at least one evolutionarily conserved Dsx binding site. These results provide new insights into the organization of the *Drosophila* sex determination pathway and how the downstream regulators Dsx and Fru cooperate to control sexual dimorphism in the gonad and brain.

## Results

### Male-specific fruitless expression in the testis

To examine Fru expression in the gonad, we used the anti-Fru^Com^ antibody that recognizes all Fru isoforms [14]. Interestingly, we found that Fru has a dynamic and male-specific pattern of expression within the developing gonad. While the gonad forms during embryogenesis and the hub and cyst stem cells are specified in the late embryo and early L1 stage [17,22], no anti-Fru immunoreactivity was observed in the gonads of either sex at these times (S1A–S1B’ Fig). Fru expression was first observed in some late L2 stage male gonads (S1C–S1D’ Fig) but was only consistently observed in L3 stage gonads (Figs 1C and S1E–S1F’). In the 3^rd^ instar larval (L3) stage, we observed Fru immunoreactivity in the hub cells (co-stained for Armadillo, Arm) and in cyst stem cells of the male GSC niche and the early cyst lineage (Traffic jam, Tj, Fig 1C). Within the ovary, we did not observe Fru expression in the apical cap from which the terminal filaments will form, or in the Tj-positive somatic cells that are intermingled with the germ cells at this stage (Figs 1D and S1E). Occasionally, we detected weak Fru signal in the basal epithelium of the ovary. We did not observe Fru expression in the germ cells (Vasa-positive) of either sex. This male-specific expression pattern is maintained in the adult GSC niche where we observed Fru colocalizing with Tj-expressing hub cells, cyst stem cells and early cyst cells (Fig 1E). In contrast, Fru is not expressed in the terminal filament cells or the Tj-expressing somatic cells of the germarium (Fig 1F).

Tra-mediated alternative splicing of *P1 fru* transcripts is the only mechanism that is known to generate male-specific Fru expression. However, *P1* expression was not detected in the male reproductive system by northern blot [13]. To test if Fru proteins detected by the anti-Fru^Com^ antibody were from the *P1* transcript, we utilized an engineered *fru* allele, *fru*^*F*^, which generates female-spliced transcripts from *P1* in both sexes [23]. These transcripts do not encode functional Fru protein and lack the anti-Fru^Com^ antibody epitope, while other *fru* transcripts remain intact. If male-specific Fru expression in the gonad is due to sex-specific splicing of *P1*, the anti-Fru^Com^ immunoreactivity should be abolished in the *fru*^*F*^ mutant testes. However, we observed normal Fru^Com^ expression in *fru*^*F*^ mutant adult testes, suggesting that *P1*-derived *fru* transcripts are not responsible for male-specific Fru expression (S1G Fig). Consistent with this, flies carrying a modified *fru* locus expressing *Gal4* in place of the *P1* transcripts (*fru*^*Gal4*^, [24]) did not exhibit any Gal4 activity in the testis tip when combined with a *UAS-mCD8GFP* reporter (S1H Fig). To determine which promoter drives *fru* expression in the male GSC niche, we generated cDNA from L3 stage testes that lack innervation by the *fru*^*M*^-expressing neurons [25]. RT-PCR conducted with promoter-specific primers showed that transcripts generated from the *P3* and *P4* promoters were expressed whereas *P1* and *P2* transcripts were not detected in the gonad (Fig 1G). Fru proteins contain one of four alternative zinc finger (ZnF) domains (A, B, C, or D) located at the C-terminus of the mature protein (Fig 1A). These Fru isoforms have distinct DNA binding motifs and play isoform-specific roles in the CNS [26]. Interestingly, testes mutant specifically for the B isoform of Fru (*fru*^*ΔB*^*/fru*^*Sat15*^) exhibit greatly reduced immunoreactivity for Fru^Com^ (S1I–S1J’ Fig) whereas we found no significant reduction in the Fru^Com^ level when *fru*^*ΔA*^ and *fru*^*ΔC*^ mutant gonads were examined (S1K–S1M’ Fig), indicating that either Fru^B^ is the major Fru isoform in the testis or it is required for expression or stability of other isoforms.

We conclude that Fru is expressed sex-specifically in the male somatic gonad, specifically in the region of the gonad stem cell niche, and that this expression is independent of the *P1* promoter, the only known promoter subject to sex-specific alternative splicing.

### Male-specific Fru expression is dependent on *dsx* and independent of alternative splicing by Tra

**Fig 2:**
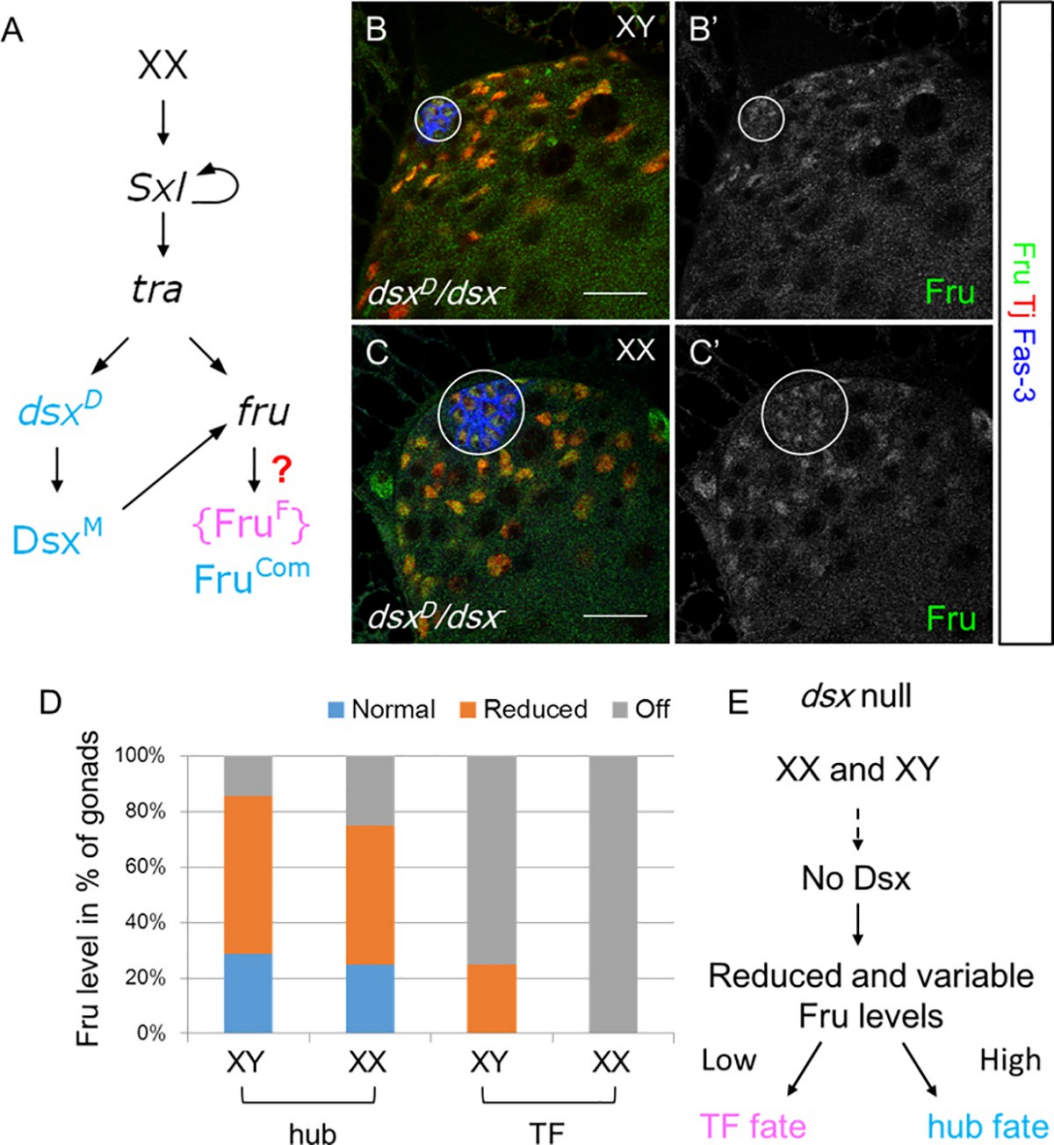
Dsx is necessary and sufficient for sex-specific Fru^Com^ expression. (A) Schematic of the experiment setup for (B-G). Under current thinking, *fru* is only regulated sex-specifically through alternative splicing by Tra. Males have default splicing and make the FruM protein, while Tra in females generates a transcript that produces the small FruF peptide that is thought to have no function. By using an allele of *dsx* that makes the male form of the Dsx protein in females, we create a situation where animals have the male form of Dsx but also express the female Tra protein. Thus, if *fru* is being regulated by Tra, it should respond in the female mode and be off in the gonad, but if *fru* is regulated by Dsx, it should respond in the male mode and be expressed. (B-C) Late L3 stage XX and XY gonads. Circle denotes the hub. Scale bars represent 20 μm. (D) Distribution of Fru expression level detected by the anti-Fru^Com^ antibody in *Df(3R)dsx*^3^*/dsx*^*1*^ gonads of the late L3 stage. Sample sizes are 4, 8, 12 and7 respective to the order presented in the bar graphs. (E) A model summarizing the Fru expression level in *dsx* null gonads.

Since our previous genomic analyses indicated that *fru* is a candidate Dsx target gene [15], we considered the possibility that sex-specific Fru expression in the gonad is regulated at the transcriptional level by Dsx. Normally, Tra acts to splice both *dsx* and *P1*-derived *fru* into their female-specific isoforms. To test whether male-specific Fru expression is dependent on *dsx* instead of *tra*, we utilized a genetic background that expresses the active (female) form of Tra but the male form of Dsx. This test utilizes an allele of *dsx* that can only produce the male isoform, even in XX animals (*XX; dsx*^*D*^*/Df(3R)dsx*^*3*^, Fig 2A). In this test, if the sex-specific expression of Fru in the gonad is dependent on female-specific splicing by Tra, or other components of the sex determination cascade upstream of *dsx*, we would expect Fru to be regulated in the “female mode” and not be expressed in the gonad. In contrast, if Fru expression is regulated by Dsx, we would expect Fru to be expressed in the “male mode” in the stem cell niche similar to wild-type testes. In *XX; dsx*^*D*^*/Df(3R)dsx*^*3*^ animals, we found that a male niche formed (Fig 2C). Further, we observed robust and consistent Fru expression in L3 stage gonads, which overlapped with Fasciclin-3 (Fas-3) and Tj in the hub cells and the early CySC lineage, and was indistinguishable from the XY siblings (Fig 2B–2C’). This result indicates that Fru expression in the gonad is dependent on *dsx* and independent of *tra*.

We then wanted to determine the expression of Fru in the gonad in the absence of *dsx* function. Dsx^F^ and Dsx^M^ can often bind to the same target genes but regulate gene expression in opposite directions [27–30]. Therefore, we predicted that Dsx^M^ activates Fru expression in the testis while Dsx^F^ represses Fru expression in the ovary, and that loss of *dsx* would cause Fru to be expressed at an intermediate level in both XX and XY gonads. In *dsx* mutants, half of both XX and XY gonads remain as hubs, while the other half switch to form TFs during the L3 stage. As a result, either a hub or TFs can be found in both XX and XY gonads [21]. We examined Fru expression levels in late L3 *dsx* null gonads and categorized the results by chromosomal sex and niche fate (hub vs. TFs, Fig 2D). Indeed, we found that *dsx* mutant gonads expressed Fru at an intermediate level, but that the level was highly variable (S2A–S2D’ Fig). Further, the level of Fru expression correlated with whether the gonads had male or female niche structures: gonads with TFs were less likely to express Fru in the apical cap and TFs, while gonads with hubs tended to have higher levels of Fru expression.

Taken together, these findings indicate that sex-specific Fru expression in the gonad is regulated by *dsx*, and Dsx^M^ is required for robust and consistent Fru expression in the male niche while Dsx^F^ is required to repress Fru expression in the female niche. Further, the level of Fru expression in *dsx* mutants correlated with whether the gonad developed a male or female niche (Fig 2D and 2E). While we don’t know what regulates the variable level of Fru expression in the absence of *dsx*, this correlation suggests that *fru* influences male niche identity.

### *fru* functions downstream of *dsx* to maintain the male niche during development

**Fig 3:**
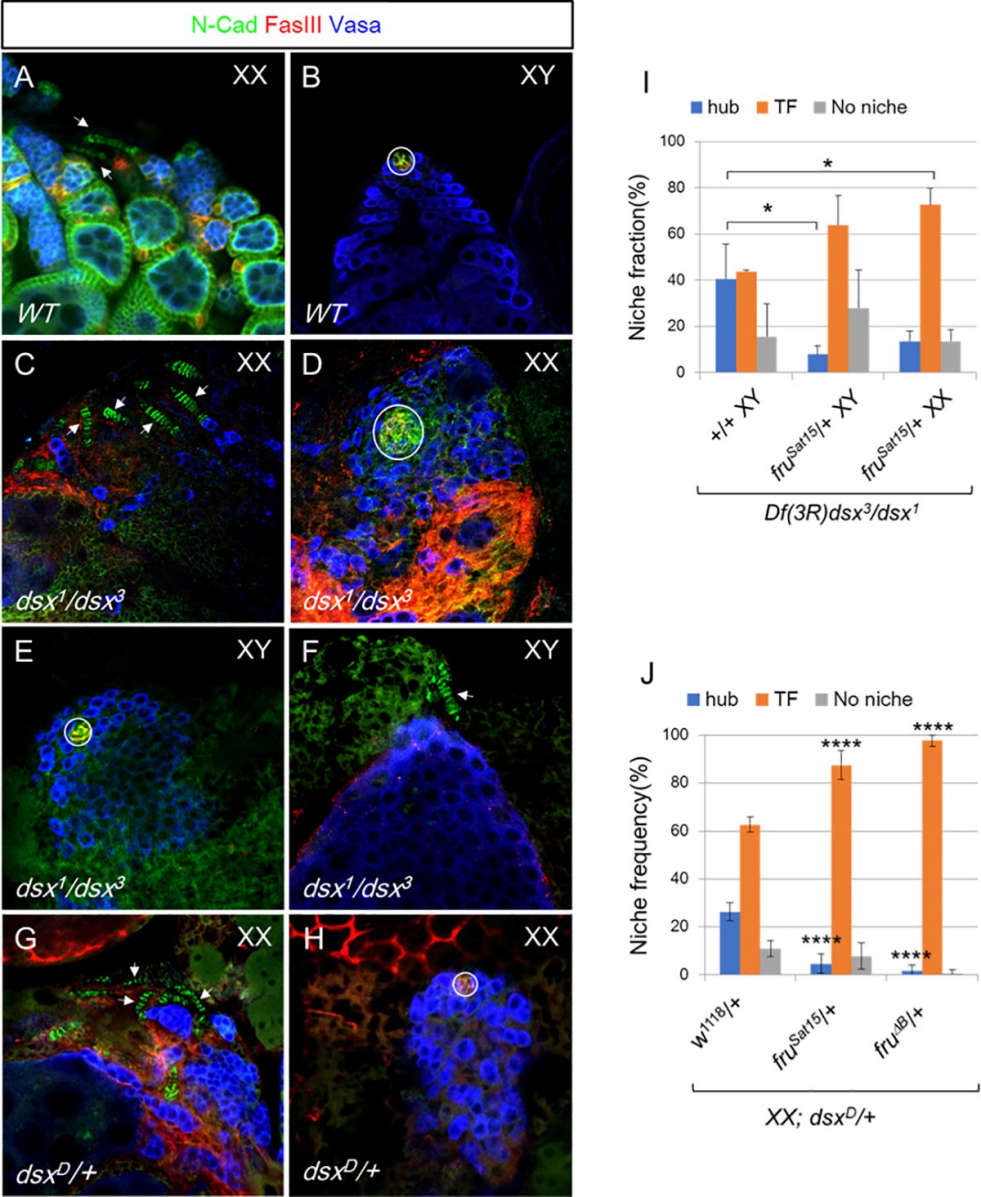
*fru* genetically interacts with *dsx* to maintain the male niche identity of *dsx* mutant gonads. (A-H) Representative gonad morphology and niche identity in wildtype (A,B) or *dsx* mutant (C-H) animals. Genotype as indicated. Hubs indicated by circles; TFs indicated by arrows. Note that the niche identity could be either a hub or TFs in *XX; dsx*^*1*^*/dsx*^*3*^, *XY;dsx*^*1*^*/dsx*^*3*^ *or XX; dsx*^*D*^*/-* animals. (I) Quantification of niche identity in 1–2 days old *Df(3R)dsx*^*3*^*/dsx*^*1*^ flies in the control background or with *fru* alleles. (J) Quantification of niche identity in 1–2 days old *XX*; *dsx*^*D*^*/+* flies in the control background or with *fru* alleles. *, p < 0.05; ***, p < 0.001; ****, p < 0.0001; ns, p> 0.05. Refer to S1 Table for sample sizes.

The fact that some *dsx* mutant gonads switch from having hubs to TFs during the L3 stage, at the time that the female niche normally develops, indicates that *dsx* is normally required in male gonads to maintain the male fate [21]. Fru is not expressed in the testis at the time of male niche formation during embryogenesis, but Fru expression initiates at the L2/L3 stage at the time that male niches must maintain hub fate, suggesting that Fru may be important for hub maintenance. We reasoned that if a higher Fru expression level is needed in *dsx* mutant gonads to maintain the hub identity or prevent TF formation, decreasing Fru levels by removing one copy of *fru* would be sufficient to “tip the balance” and cause more gonads to switch to the formation of TFs. Conversely, if Fru expression is only a consequence of male-specific cell fate, changing Fru expression level would not alter the chances of a *dsx* mutant gonad developing a hub or TFs. As previously reported [21], *dsx* mutant gonads had a roughly equal chance of forming hubs or TFs (with another fraction forming no discernable niche structure, Fig 3C–3F). When one copy of *fru* was removed in this genetic background (*dsx*^*1*^*/Df(3R)dsx*^*3*^, *fru*^*Sat15*^*/+*), we observed that the fraction of XY gonads with hubs decreased while the fraction that formed TFs increased (Fig 3I). XX animals showed a similar shift towards the TF fate. A similar assay was conducted using the *dsx*^*D*^*/+* genetic background, where Dsx^M^ and Dsx^F^ are simultaneously expressed in XX individuals and interfere with one another, thus causing these animals to develop similar to *dsx* null animals [15] (Fig 3G and 3H). In *XX*; *dsx*^*D*^*/+* adults, we again observed a shift from hubs to TF fate in the presence of either one copy of a *fru* null allele (*fru*^*Sat15*^*/+*) or an allele specific null for *fru*^*B*^ (*fru*^*ΔB*^*/+*) (Fig 3J). These results suggest that, in *dsx* mutants, *fru* is required to maintain the hub fate and inhibit the TF fate.

### Loss of *fru* is not sufficient to cause gonad sex reversal

We next wanted to know whether loss of *fru* alone could cause gonad sex reversal. *fru* null and *fru*^*ΔB*^ mutant flies all die in pupal stages [26], soon after the L3 stage when male niche fate must be maintained. We observed no morphological defect in the hub prior to lethality (S3A–S3D Fig), suggesting that loss of *fru* alone was not sufficient to cause a loss of hub fate. Clonal analysis using null alleles of *fru* is not possible in the hub as these cells are post-mitotic from mid-embryogenesis onwards. To determine whether *fru* helps to maintain the male niche in adult testes, we performed cell-type specific RNA-interference (RNAi)-mediated knockdown of *fru*. Knockdown of *fru* in the hub using the *upd-Gal4* driver did not yield a hub phenotype (S3E–S3F’ Fig). Knockdown of *fru* in the CySC lineage using the *tj-Gal4* also did not cause these cells to take on female morphology (S3G and S3G’ Fig). Thus, either the loss of Fru activity is not sufficient to cause testis sex reversal or the RNAi knockdown was insufficient to induce this phenotype. It is worth noting that when testes were examined 2 weeks after eclosure we did observe an expansion of Tj-positive cyst cells in *tj>fru RNAi* testes compared to *tj>control RNAi* testes (S3H–S3J Fig), suggesting that *fru* has functions in regulating CySC lineage differentiation. However, since we observed no switching from hub to TF fate in *fru* mutants, it is likely that *dsx* regulates other targets in addition to *fru* to promote hub maintenance.

### *fru* is cell-autonomously required for cyst stem cell maintenance

**Fig 4:**
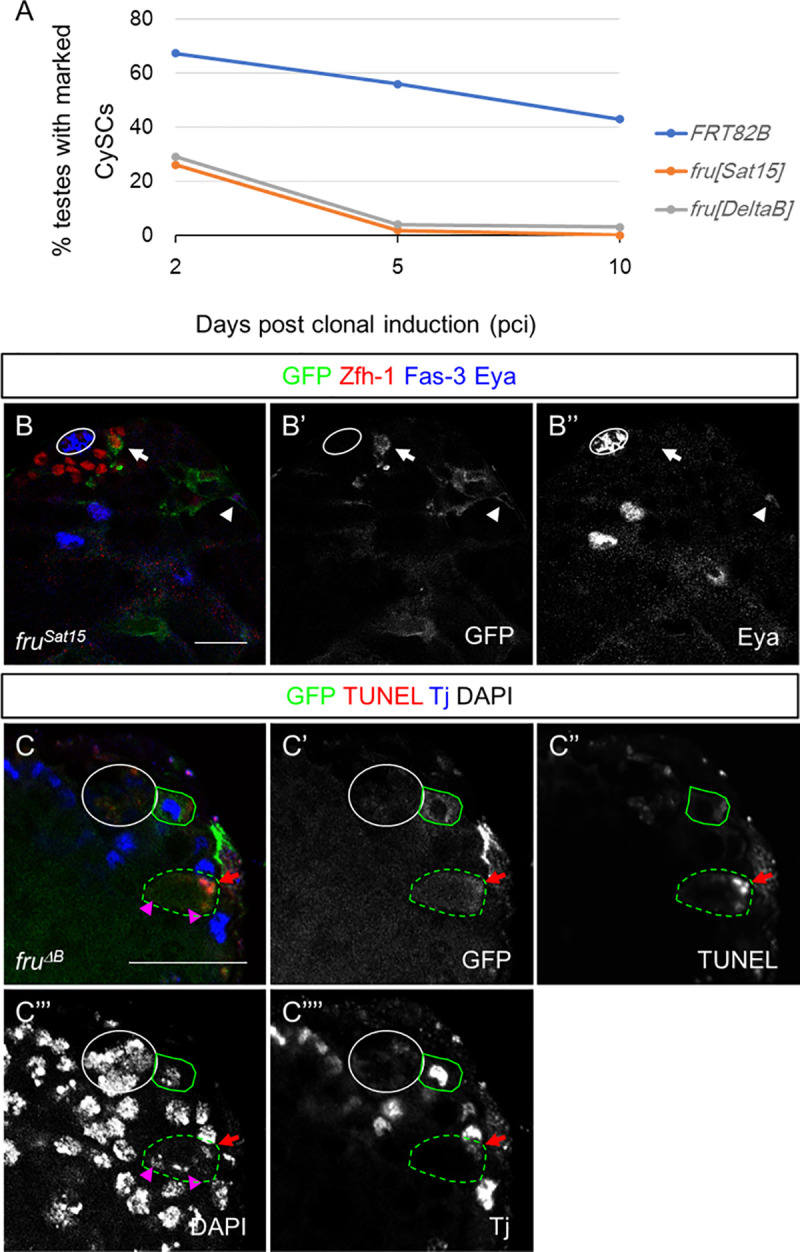
*fru* is cell-autonomously required for cyst stem cell maintenance and cyst cell survival. (A) The percentage of control (*FRT82B*) and *fru* mutant (*fru*^*Sat15*^ and *fru*^*ΔB*^) CySC clones maintained at the niche post clonal induction (pci). (B-C) Immunolabeling of adult testis with clones of cells of indicated genotypes labeled with GFP. (B) A representative image at 4 days pci showing Zfh-1 and Eya expression in *fru*^*Sat15*^ CySC and cyst cell clones. Arrow denotes CySC clone and arrowhead denotes cyst cell clone. (C) A representative image at 4 days pci showing *fru*^*ΔB*^ cyst cell (circled with dashed green line) rather than CySC (circled with solid green line) was positive for TUNEL. Red arrow denotes *fru*^*ΔB*^ cyst cell clone; magenta arrowheads denote germ cells encapsulated by the dying *fru*^*ΔB*^ cyst cell clones with diminished DAPI staining. n = 22. Circle denotes the hub. Scale bars represent 20 μm.

To investigate further *fru’s* function in the CySC lineage, we generated *fru*-mutant clones that were positively marked with GFP using the MARCM technique [31] and asked if CySC clones could be generated and maintained. Control (*FRT82B*) CySC clones were observed in 67% (n = 61), 56% (n = 129) and 43% (n = 56) of the testes examined at 2, 5, and 10 days post clone induction (pci), respectively (Fig 4A and S2 Table). In contrast, CySC clones homozygous mutant for *fru*^*Sat15*^ were observed less frequently at 2 days pci (26%, n = 46), lost rapidly by 5 days pci (1.8%, n = 113), and were completely absent by 10 days pci (0%, n = 78). *fru*^*ΔB*^ mutant CySCs were also observed at a low frequency at 2 days pci (29%, n = 55), and were lost at a similar rate as *fru*^*Sat15*^ clones (5 days pci:4%, n = 101; 10 days pci: 3%, n = 66). These results indicate that *fru* is required for CySC maintenance.

We next investigated the mechanism by which *fru* regulates the CySC lineage. Two possible explanations of CySC loss are precocious differentiation and CySC cell death. Zfh-1 is expressed in CySCs and early differentiating cyst cells, while Eyes absent (Eya) is only expressed in later stages of cyst cell differentiation. In *fru* mutant clones at 2–4 days pci, the somatic cells closest to the hub still expressed Zfh-1 and did not express Eya, indicating they were not prematurely differentiating (Fig 4B). Similarly, *fru* mutant CySC did not exhibit signs of DNA fragmentation characteristic of apoptosis (TUNEL assay, Fig 4C). These results indicate that *fru* is required for CySC maintenance in a manner not due to premature differentiation or CySC death. However, we did observe that 45% (n = 22) of testes with *fru*^*ΔB*^ cyst cell clones had TUNEL-positive, *fru-*mutant cyst cells, which was not observed in testes carrying control cyst cell clones (0%, n = 8), suggesting that *fru* may function in later cyst cell survival in addition to CySC maintenance.

### Ectopic expression of Fru inhibits terminal filament formation and partially masculinizes the female niche

**Fig 5:**
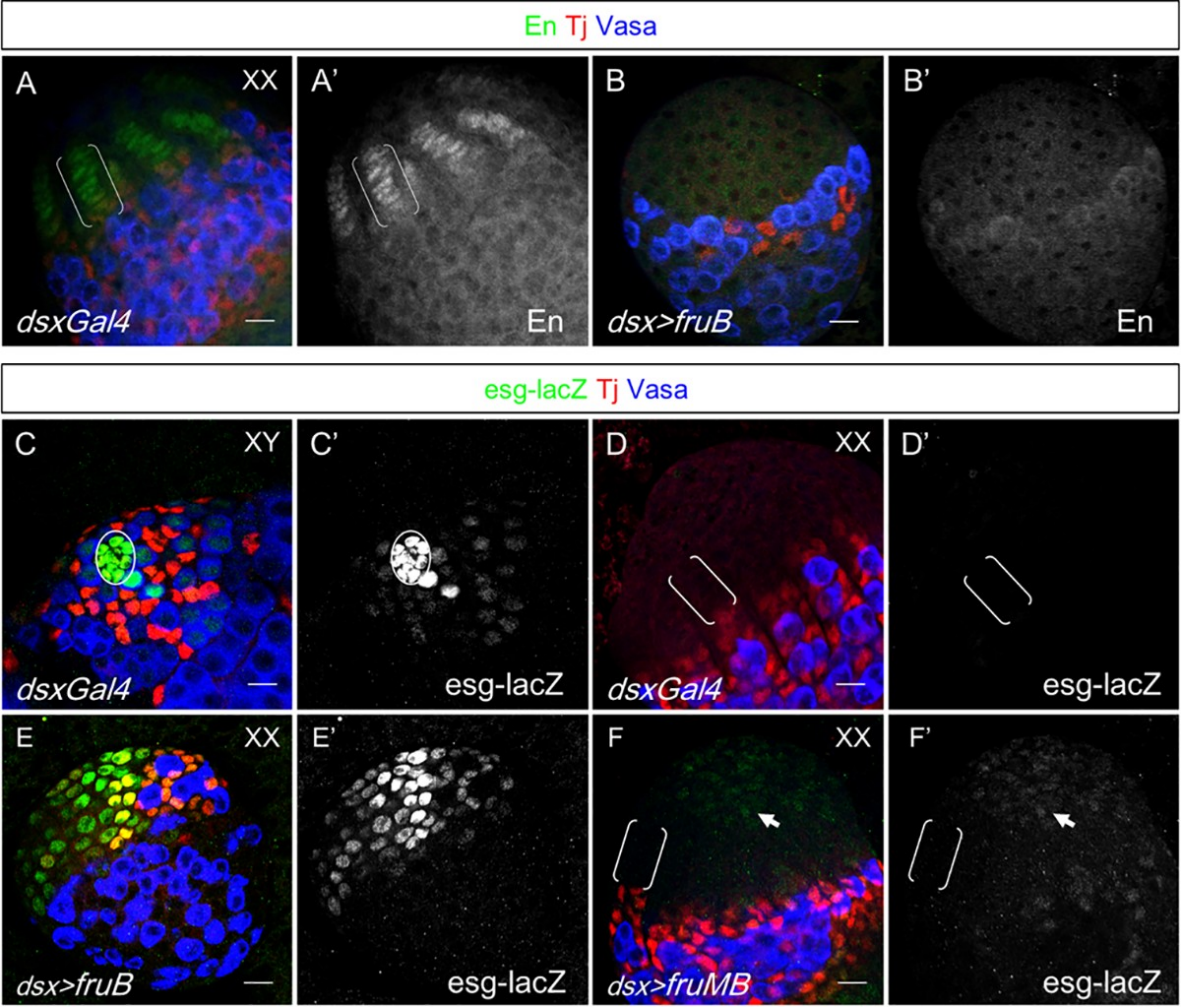
Fru overexpression inhibits terminal filament development and masculinizes the female niche. Gonads of the white prepupal stage (WPP). Genotype, antibodies used and chromosomal sex as indicated. Circle denotes the hub; brackets denote the TF. (A,B) En labels the developing TF of the wt ovary (A) but is suppressed by ectopic expression of FruB (B). (C-F) esg-lacZ is strongly expressed in the control testis hub (C) but not in the control ovary (D). Ectopic esg-lacZ expression is induced by expression of FruB (E), but not by the *fru* P1 derived isoform FruMB (F). Arrows point out weak apical cap expression in F, F’. Scale bars represent 20 μm.

Though *fru* is not necessary for hub maintenance, we next asked whether *fru* is sufficient to cause defects in normal female niche development. We expressed the Fru^B^ (*UAS-fruB*) isoform [32] in *dsx-*expressing cells of the developing ovary using *dsx-Gal4* [33] (S5A–S5B’ Fig). Engrailed (En) is a TF-specific marker and is required for specification of TF cells from the apical cap [34]. When white prepupae (WPP) were examined, control ovaries lacking the *UAS* transgene all had groups of 6–8 disc-shaped, En-expressing cells aligning at the base of the apical cap (n = 7) (Fig 5A). In contrast, ovaries expressing Fru^B^ failed to robustly express En or intercalate En-expressing cells into filaments (n = 25) (Fig 5B). To determine if Fru^B^ overexpression masculinized the female niche, we examined the male-specific niche marker, *escargot (es*g), with an enhancer trap (*esg*^*M5-4*^) that reports *esg* activity through the expression of β-Galactosidase [17,35]. We observed strong expression of *esg-lacZ* in the hub of control testes (n = 6), and no expression throughout the control ovary (Fig 5C–5D). In the WPP stage, ovaries ectopically expressing Fru^B^ had a high level of esg-LacZ in the apical cap region (n = 23) (Fig 5E). However, we did not observe any evidence for the formation of hubs in these gonads. Proteins produced from *fru P1* promoter in males (Fru^M^) have an N-terminal domain not found in Fru proteins derived from other promoters. Interestingly, ectopic expression of the B isoform of Fru^M^ (Fru^MB^) in the developing ovary did not inhibit TF formation and only induced weak esg-lacZ expression in the apical cap (Fig 5F, arrow). This indicates that Fru^Com^ has a stronger masculinizing effect in the gonad than Fru^M^. Overall, we conclude that overexpression of Fru^B^ is sufficient to interfere with ovary development and partially masculinize somatic cells, but it is not, by itself, sufficient to induce hub formation.

### An evolutionarily conserved Dsx binding site is required for normal *fru* expression in hub cells

**Fig 6:**
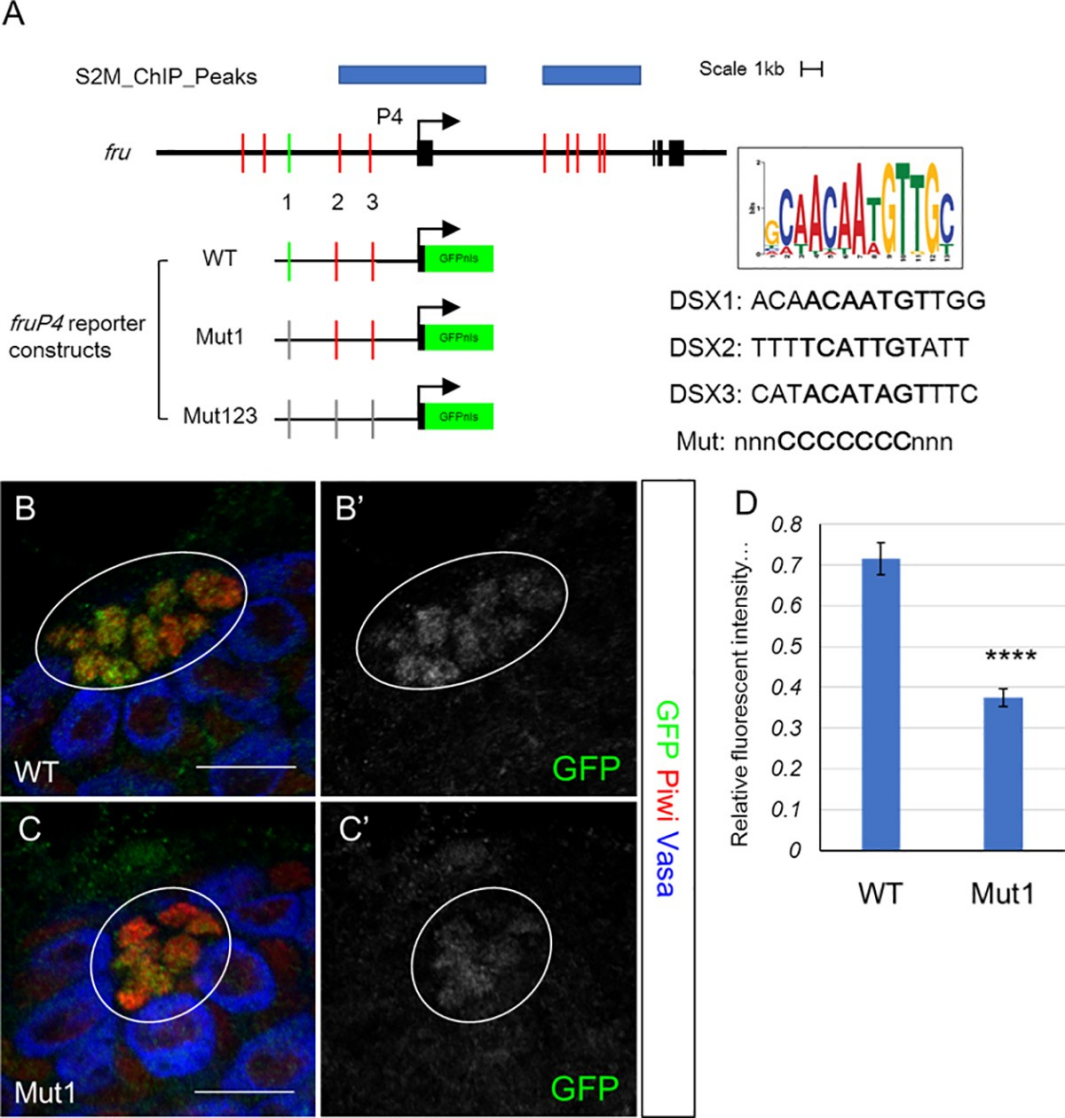
A canonical Dsx binding site upstream of *fru P4* is required to maintain normal *P4* expression level in the hub cells. (A) Schematic diagram of the *fru P4* enhancer-promoter constructs. The position of Dsx occupancy (blue), and Dsx binding motifs (top 10% PWM, red and green bars) relative to the *P4* promoter are shown. The Dsx consensus motif and the sequences of DSX1, DSX2, and DSX3 are shown. (B-C) Representative images of GFP expression levels in late L3 stage testes carrying WT and Mut1 constructs. Scale bars represent 20 μm. Circle denotes the hub. (D) Quantification of GFP relative fluorescent intensity per hub cell in WT and Mut1 testes. Data are presented as Mean ± SEM. WT, n = 125; Mut1, n = 115. Student’s t-test.

Previously, we have used a combination of whole-genome Dsx occupancy data, sequence searches for biochemically and genomically defined Dsx binding sites, and evolutionary conservation of these sites across sequenced Drosophila species, to identify likely Dsx targets in the genome [15]. This work indicated that *fru* was a candidate for direct regulation by Dsx, with the regions around the *P3* and *P4* promoters being particularly likely to contain Dsx-responsive elements (S4A–S4D Fig). We identified a Dsx motif (DSX1) 6.3 kb upstream of *P4* which is completely conserved across 21 *Drosophila* species, is a perfect match to the Dsx core binding motif (ACAATGT, [27,36]), and also matched surrounding nucleotides that may be important for Dsx binding [37] (Figs 6A and S6A). A transgenic reporter was created in which a 7.5 kb genomic sequence including DSX1 and the *P4* promoter was placed upstream of a nuclear GFP reporter (WT reporter, Fig 6A). Transgenic flies carrying this construct (WT) expressed GFP in the hub, but not in the CySC or cyst cells, and expression was also not observed in the ovary (Figs 6B and S6B). Based on what we know about regulation of the few Dsx targets that have been studied, sex-specific expression in a given tissue requires both tissue-specific control elements and Dsx-responsive elements. Thus, it is not surprising that the WT *fru* reporter would be expressed in only a subset of Fru-expressing cells in the testis.

To test if DSX1 is essential for proper sex-specific expression of *fru*, we created the Mut1 reporter construct where the 7 core nucleotides of DSX1 are replaced by G nucleotides. When GFP expression level in the hub was quantified and compared between transgenic flies containing WT and Mut1 constructs (see Method for details), we found that the GFP fluorescence intensity in hub cells of the Mut1 reporter was significantly decreased relative to the wild-type reporter (p<0.0001, student t-test) (Fig 6B–6D). However, we did not observe GFP expression in females which would have been expected if Dsx^F^ acts as a repressor of *fru* in the ovary (S6C Fig). This is not surprising given the low level of Fru expression we observed in *dsx* mutants that formed female niche structures. Two other sites within the reporter transgene more weakly resemble the Dsx consensus motif, but are divergent in the 7-nucleotide core region (DSX2 and DSX3, Fig 6A). Mutation of these sites (Mut123) did not further decrease GFP expression in the hub or lead to GFP expression in the ovary (S6D and S6E Fig).

Collectively, these results support that *fru* is a direct target gene of Dsx. The conserved DSX1 motif is needed for robust expression in hub cells, but additional Dsx binding sites present in the *fru* locus, as well as additional tissue-specific elements, are likely needed to completely recapitulate sexually-dimorphic Fru expression in the gonad.

## Discussion

Over the past decades, much effort has been focused on understanding the functions of *fru* in regulating sex-specific behaviors, yet it remained unclear whether *fru* plays a role in regulating sexual dimorphism outside the nervous system. The work presented here demonstrates that Fru is expressed male-specifically in the gonad stem cell niche, and is required for CySC maintenance, cyst cell survival, and for the maintenance of the hub during larval development. Further, male-specific expression of Fru in the gonad is independent of the previously described mechanism of sex-specific alternative splicing by Tra, and is instead dependent on *dsx*. *fru* appears to be a direct target for transcriptional regulation by Dsx. This work provides evidence that *fru* regulates sex-specific development outside the nervous system and alters traditional thinking about the structure of the *Drosophila* sex determination pathway.

### *fru* function outside the nervous system

While it was previously reported that *fru* is expressed in tissues other than the nervous system, including in the gonad [13], a function for *fru* outside the nervous system was previously unknown. We find that Fru is expressed in the developing and adult testis in the hub, the CySC, and the early developing cyst cells. Importantly, we find that *fru* is important for the proper function of these cells.

Fru is not expressed at the time of hub formation during embryogenesis, but expression is initiated during the L2/L3 larval stage. This correlates with a time period when the hub must be maintained and resist transforming into female niche structures; in *dsx* mutants, all gonads in XX and XY animals develop hubs, but in half of each, hubs transform into terminal filament cells and cap cells [21]. *fru* is not required for initial hub formation, consistent with it not being expressed at that time. *fru* is also not, by itself, required for hub maintenance under the conditions that we have been able to assay (prior to the pupal lethality of *fru* null mutant animals). However, under conditions where hub maintenance is compromised by loss of *dsx* function, *fru* clearly plays a role in influencing whether a gonad will retain a hub, or transform into TF. Fru expression in *dsx* mutant gonads correlates with whether they formed male or female niche structures (Fig 2D), and removing even a single allele of *fru* is sufficient to induce more hubs to transform into TFs (Fig 3). Finally, ectopic expression of Fru in females is sufficient to inhibit TF formation and partially masculinize the gonad (Fig 5B and 5E), but does not induce hub formation. Thus, we propose that *fru* is one factor acting downstream of *dsx* in the maintenance of the male gonad stem cell niche, but that it acts in combination with other factors that also regulate this process.

We also demonstrated that *fru* is required for CySC maintenance and for the survival of differentiating cyst cells. Loss of *fru* from the CySC lineage led to rapid loss of these CySCs from the testis niche (Fig 4A). Since we did not observe precocious differentiation of CySCs or an increase in their apoptosis (Fig 4B and 4C), these mechanisms do not appear to contribute to CySC loss. One possibility is that *fru* is needed for CySCs to have normal expression of adhesion proteins and compete with other stem cells for niche occupancy. It has been shown that *fru* regulates the Slit-robo pathway and *robo1* is a direct target of *fru* in the CNS [8,38]. Interestingly, the Slit-Robo pathway also functions in the CySCs to modulate E-cadherin levels and control the ability of CySCs to compete for occupancy in the niche [39]. Therefore, *fru* may use similar mechanisms to maintain CySC attachment to the hub. *fru* also influences survival in the differentiating cyst cells, as we observed an increase in cell death in these cells in *fru* mutants. Several reports have demonstrated that *fru* represses programmed cell death in the nervous system [5,7,40]. It was further indicated that the cell death gene *reaper* is a putative target of Fru [26]. Thus, *fru* may play a role in repressing the apoptosis of cyst cells.

In summary, *fru* function is clearly important for male niche maintenance and the function of the CySCs and their differentiating progeny. This provides clear evidence that *fru* regulates sex-specific development in tissues other than the nervous system. Whether additional tissues are also regulated by *fru* remains to be determined.

### A change in our view of the sex determination pathway

**Fig 7:**
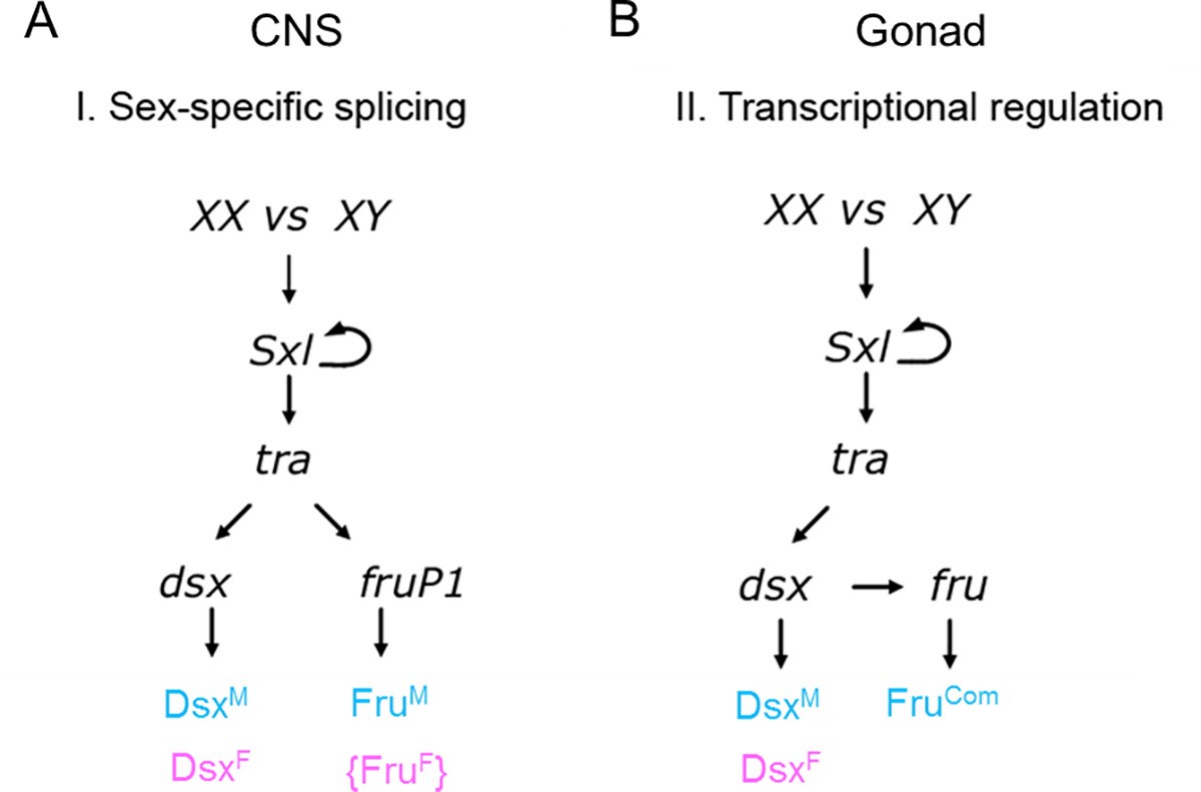
Proposed model of the *Drosophila* sex determination pathway. **(A)** The canonical sex determination pathway has *dsx and fru* as independent and parallel branches of the pathway, both regulated sex-specifically via alternative splicing by Tra. In males, default splicing produces DsxM and FruM while Tra-dependent splicing produces DsxF and the non-functional FruF peptide. This model of regulation has been observed in the CNS. (B) Our data support an alternative mechanism for sex-specific regulation of *fru*, where *fru* transcription is directly regulated by Dsx. This occurs through *fru* promoter(s) different than *fru* P1, which produce the transcript regulated by Tra. This mode of regulation occurs in the gonad and may also exist in the CNS in addition to the mechanism in (A).

Previously, it was thought that the only mechanism by which sex-specific functions of *fru* were regulated was through Tra-dependent alternative splicing of the *P1* transcripts. *fru* null alleles are lethal in both sexes and Fru proteins derived from *non-P1* promoters were thought to be sex-nonspecific and not to contribute to sex determination. Thus, *fru* and *dsx* were considered as parallel branches of the sex determination pathway, each independently regulated by Tra. Here we demonstrate that *fru* can also be regulated in a manner independent of *tra* and dependent on *dsx*, and provide evidence that *fru* is a direct target for transcriptional regulation by Dsx (Fig 7). First, Fru expression in the testis is independent of the *P1* transcript that is regulated by Tra. A *P1* Gal4 reporter is not expressed in the testis and a mutation that prevents Fru^M^ expression from *P1* does not affect Fru immunoreactivity in the testis (S1G and S1H Fig). Second, in animals that simultaneously express the female form of *tra* (Tra on) and the male form of Dsx (XX; *dsx*^*D*^*/Df(3R)dsx*^*3*^), Fru is expressed in the male mode in the testis, demonstrating that it is regulated by *dsx* and not *tra*. Finally, an evolutionarily conserved Dsx consensus binding site upstream of the *P4* promoter is required for proper expression levels of a *fru P4* reporter in the testis. Together, these data demonstrate a novel mode for *fru* regulation by the sex determination pathway, where sex-specific expression of *fru* is regulated by *dsx*. It also means that the large number of *fru* transcripts that do not arise from the *P1* promoter can be expressed in a sex-specific manner to contribute to sexual dimorphism.

The male and female forms of Dsx contain the same DNA binding domain and can regulate the same target genes, but often have opposite effects on gene expression. Prior to this study, the documented Dsx targets (*Yolk proteins 1* and *2*, *bric-a-brac* and *desatF*), along with other proposed targets, were all expressed at higher levels in females than males [27–29,41]. Thus, for these targets, Dsx^F^ acts as an activator and Dsx^M^ acts as a repressor (or Dsx^M^ has no role [41]). Interestingly, *fru* is the first identified Dsx target that is expressed in a male-biased manner. Thus, for direct regulation of *fru*, Dsx^M^ would activate expression while Dsx^F^ represses. Mechanistically for Dsx, this implies that the male and female isoforms are not dedicated repressors and activators, respectively, but may be able to switch their mode of regulation in a tissue-specific or target-specific manner. Mouse DMRT1 has also been shown to regulate gene expression both as transcriptional activator and repressor [42]. Thus, it is quite possible that bifunctional transcriptional regulation is a conserved characteristic of DMRTs.

It is possible that *dsx* regulation of *fru* occurs in the nervous system as well, where it co-exists with direct regulation of *fru* alternative splicing by Tra. It was originally thought that alternative splicing of the *fru P1* transcript by *tra* was essential for male courtship behavior [23]. However, more recently it was found that these animals could exhibit male courtship behavior if they were simply stimulated by other flies prior to testing [9]. Interestingly, the courtship behavior exhibited by these males was dependent on *dsx*. We propose that *fru* might still be essential for male courtship in these *fru P1*-mutants, but that sex-specific *fru* expression is dependent on transcriptional regulation of other *fru* promoters by Dsx.

### Evolution of the sex determination pathway

If sex-specific *fru* function can be regulated both through alternative splicing by Tra and through transcriptional regulation by Dsx, it raises the question of what is the relationship between these two modes of regulation? We propose that regulation of *fru* by Dsx is the more ancient version of the sex determination pathway and that additional regulation of *fru* by Tra evolved subsequently, through the acquisition of regulatory RNA elements in the *fru* P1 transcript. This model is supported by studies of *fru* gene structures in distantly related Dipteran species, and species of other insect orders, that illustrate the considerable variability in the organization of sequences controlling *fru* splicing [43]. Further, in some insects, no evidence for alternative splicing of *fru* has been found, yet *fru* still plays an important role in males to control courtship behaviors [44–46]. Finally, in the Hawaiian picture-winged group of subgenus *Drosophila*, the *fru* orthologues lack the *P1* promoter, and *non-P1 fru* transcripts exhibit male-specific expression [47,48], similar to what we propose for *non-P1 fru* transcripts in *D*. *melanogaster*. Thus, it appears that regulation of *fru* by *dsx* may be the evolutionarily more ancient mechanism for sex-specific control of *fru*, while Tra-dependent splicing of *P1* transcripts is a more recent adaptation. More broadly, *tra* is not conserved in the sex determination pathway in the majority of animal groups, while homologs of Dsx, the DMRTs, are virtually universal in animal sex determination. Thus, if Fru orthologs are involved in the creation of sexual dimorphism in the body or the brain in other animals, they cannot be regulated by Tra but may be regulated by DMRTs.

## Methods

### Fly strains

The following strains were used: *fru*^*W24*^ (S. Goodwin), *fru*^*Sat15*^ (S. Goodwin), *fru*^*ΔB*^ (S. Goodwin), *fru*^*Gal4*^ (S. Goodwin), *dsx*^*D*^, *Df(3R)dsx*^*3*^, *dsx*^*1*^, *dsx*^*GAL4*^ (B. Baker), *dsx-Gal4* (S. Goodwin), *UAS-fruMB* (S. Goodwin), *UAS-fruB* (S. Goodwin), *c587-Gal4* (T. Xie), *tj-Gal4* (D. Godt), *esg*^*M5-4*^ (S. DiNardo), *y^1^v1*; *P{TRiP.JF01182}attP2* (*UAS-fru*^*Com*^*-RNAi*), *yw*, *hs-FLP*, *UAS-mCD8*:*GFP; tub-Gal4*, *FRT82B*, *tub-Gal80*, *hs-FLP*, *tub-Gal4*, *UAS-GFP*.*Myc*.*nls*, *yw; FRT82B*, *tub-Gal80*, *FRT82B*, *FRT82B*, *fru*^*Sat15*^, *FRT82B*, *fru*^*ΔB*^, and *w*^*1118*^ as a control. All flies were raised at 25°C unless otherwise stated.

### Immunohistochemistry

Adult testes were dissected in PBS and fixed at room temperature for 15 minutes in 4.5% formaldehyde in PBS containing 0.1% Triton X-100 (PBTx). Adult ovaries, *dsx* mutant adult gonads, and larval gonads were dissected in PBS followed by a 10-minute fixation at room temperature in 6% formaldehyde in PBTx. Immunostaining was performed as previously described [49], and samples were mounted in 2.5% DABCO. The following primary antibodies were used: rat anti-Fru^Com^ at 1:300 (S. Goodwin); guinea pig anti-Traffic-jam (D. Godt) at 1:10,000; mouse anti-Arm N2 7A1 (DSHB, E. Wieschaus) at 1:100; chicken anti-Vasa (K. Howard) at 1:10,000; mouse anti-Fas-3 7G10 (DHSB, C. Goodman) at 1:30; mouse anti-Eya 10H6 (DSHB, S. Benzer/N.M. Bonini) at 1:25; mouse anti-Engrailed 4D9 (DSHB, C. Goodman) at 1:2; rat anti-DN-Cad DN-EX#8 (DHSB, T. Uemura) at 1:20; rabbit anti-GFP ab290 (abcam) at 1:2000; rabbit anti-Vasa (R. Lehmann) at 1:10,000; rabbit anti-Sox100B (S. Russell) at 1:1,000; rabbit anti-β-Gal (Cappel) at 1:10,000; rabbit anti-Zfh1 (R. Lehmann) at 1:5,000. Secondary Alexa 488, 546 and 633 antibodies were used at 1:500 (Invitrogen). For detection of germ cell death with Lysotracker, testes were stained with Lysotracker Red DND-99 (ThermoFisher) in PBS (1:1,000) for 30 mins before formaldehyde fixation. Immunostaining was followed as normal. For TUNEL-dependent detection of cell death, testes were fixed as normal and label with Click-iT TUNEL Alex Fluor 594 Imaging Kit (ThermoFisher) according to manufacturer’s instructions. All immunohistochemistry samples were imaged on a Zeiss LSM 700 confocal microscope.

### Developmental staging

To obtain first and second instar larvae, flies were transferred to a cage to allow egg-laying on an apple juice plate for 4 hours and were then removed. The apple juice plates were left at 25°C. Larvae were collected at desired developmental stages (36 h for mid first instar, 72 h for late second instar). Immobile third instar larvae were collected from the vials as late third instar larvae. Larvae with inverted spiracles and harden carcass were collected from vials as white prepupae.

### Genotyping and sex identification of dsx mutants

Balancer chromosomes containing a *P{Kr-Gal4*, *UAS-GFP}* transgene were used to distinguish transheterozygous *dsx* or *fru* mutant larvae from heterozygous siblings. Sex chromosome genotype of *dsx* null mutants was identified using a *P{Msl-3-GFP}* (J. Sedat) transgene, or Y chromosome marked with *Bs* (*Dp(1;Y)B*^*S*^). *XX*; *dsx*^*D*^*/+* and *XX; dsx*^*D*^*/Df(3R)dsx*^*3*^ mutants were distinguished from their XY siblings by abnormal gonad morphology.

### Quantification of niche identity

Adult flies less than 2 days old were dissected and stained with antibodies against DN-Cad, Fas-3, and Vasa, and cell nuclei were visualized via DAPI staining. Z-stack images were taken with a Zeiss LSM 700 confocal microscope with a 40x objective. The hub was defined as a compact cluster of DAPI bright somatic cells that coexpressed N-Cad and Fas-3 and were surrounded by a rosette of Vasa-positive germ cells. TFs were determined by ladder-shaped N-Cad staining around stacks of disc-shaped somatic nuclei indicated by DAPI staining. A gonad was defined as having no niche when neither TFs nor a hub was identified.

### Clonal analysis

Flies of the following genotype were used for MARCM: *hs-FLP*, *UAS-mCD8*:*GFP/Y; tub-Gal4*, *FRT82B*, *tub-Gal80/FRT82B* (control); *hs-FLP*, *UAS-mCD8*:*GFP/Y; tub-Gal4*, *FRT82B*, *tub-Gal80/FRT82B*, *fru*^*Sat15*^; *hs-FLP*, *UAS-mCD8*:*GFP/Y; tub-Gal4*, *FRT82B*, *tub-Gal80/FRT82B*, *fru*^*ΔB*^. Newly eclosed adult males (0–2 days old) were collected at 25°C prior to heat shock. Flies were heat-shocked at 37°C for 1 hour and returned to 25°C and raised in fresh vials with yeast paste. Control and mutant clones were analyzed at the indicated time points post clonal induction. CySC clones were counted as GFP-marked Zfh-1- or Tj-positive cells within one germ cell diameter to the hub and directly contacting the hub with cytoplasmic extension as indicated by mCD8:GFP. The remaining GFP marked Zfh-1- or Tj-positive cells were considered as cyst cell clones.

### RT-PCR

100 late 3^rd^ instar larval gonads were dissected into ice-cold PBS and cDNA was prepared following manufacturers’ protocols (Zymo Research Quick-RNA Miniprep Kit and Invitrogen Superscript III Kit). PCR was performed on cDNA using the following intron-spanning primer pairs (given in the 5’-3’ orientation):

RP49-F—CCGCTTCAAGGGACAGTATCTG
RP49-R—ATCTCGCCGCAGTAAACGC
TJ-F- ACCAGTGGCACATGGACGAA
TJ-R—CGCTCCCGAAGATGTGTTCA
Fru-P1-F—CGGAAAAGGGCGTATGGATTG
Fru-P1-R—TGTGCCAGTCAGCCTCTG
Fru-P2-F—AGCACGCCGGTCAAATTTG
Fru-P2-R—TCGCTCGGTTTTAGTTTCCCA
Fru-P3-F—GCACGTTCTCAGTTTGGAATTC
Fru-P3-R—CAACGAAAACCGTGAACTGTG
Fru-P4-F—GAATTGCTGGTCCATCGCTC
Fru-P4-R—GCAACTGAACCCAACTGTACC
Fru-Com-F—ATTACTCGGCCCACGTCC
Fru-Com-R—CTGCCCATGTTTCTCAAGACG

Each primer pair was validated for efficacy using whole fly cDNA from an adult male.

### Fru reporter constructs and transgenes

To generate the WT *fruP4* enhancer-promoter reporter construct, a 7.5 kb genomic sequence from *fru* genomic clone BACRP98-2G21 (BACPAC Resources Center) was amplified with the following primers (given in the 5’ to 3’ orientation) and cloned into the pJR16 vector (R. Johnston) between the BamHI and PstI site.

Fru-P4-8K-WT-F—CGGGATCCGCAACCCGTCCGTATC
Fru-P4-8K-WT-R—CAACTGCAGTGTGGGTATGGGCAAATTGA

Site-directed mutagenesis of DSX sites was performed according to the manufacturer’s protocol (NEB Q5 Site-Directed Mutagenesis Kit). The following primer sets were used:

DSX1mut-F—GGGTGTGTTAATTTGCCAGG
DSX1mut-R—CCCCTGGCTCATTAACAGACCAAT
DSX2mut-F—GGGATTTATTGCACAGGTTG
DSX2mut-R—CCCCAAATGTTAGAAAACCAAGCATTTTT
DSX3mut-F—GGGTTCTGTAATAGATAATTCAGTTC
DSX3mut-R—CCCCATGAGTAACTTCTGTGC

Transgenic flies were generated via PhiC31 integrase-mediated transgenesis. The constructs were integrated into the same genomic location (P{CaryP}attP40 on Chromosome II).

### Imaging and quantification of GFP expression in the hub

Z-stack images of the hub were taken using the same setting on a Zeiss LSM 700 confocal microscope with a 63x objective. Quantification of GFP fluorescent intensity was performed in Fiji software (ImageJ). For each gonad, five random hub cells were sampled, and background signal was sampled from a 16-cell-stage germ cell. A circle of the same size was drawn as the sample area. Average fluorescence intensity of GFP and Piwi was acquired. The relative fluorescent intensity was measured as (GFP[hub]-GFP[background]) / (Piwi[hub]-Piwi[background]).

## Supporting information

S1 Fig Immunostaining as indicated in figure. Anti-Vasa labels the germline, Anti-Arm labels the hub, anti-Tj labels the CySC and early cyst cells of the testes along with the somatic cells intermingled with germ cells in the ovary. (A-B) Wildtype L1 stage female and male gonads with no Fru expression. (C-D) Wildtype L2 stage gonads showing weak Fru expression in the hub cells and early CySC lineage of the testis. (E-F) Wildtype late L3 stage gonads showing robust Fru expression in the male GSC niche and no Fru expression in the female GSC niche (G) A representative *fru*^*F*^*/fru*^*W24*^ adult testis showing normal Fru expression in the niche. (H)A representative *fru*^*Gal4*^*>mCD8*:*GFP* testis showing no GFP expression in the niche. (I-J) Late L3 stage wildtype (I) and *fru*^*ΔB*^*/fru*^*Sat15*^ (J) gonads showing the reduced Fru^Com^ immunoreactivity in the hub and the Tj+ cyst cells (arrow). (K-M) Late L3 stage wildtype (K) and *fru*^*ΔA*^*/fru*^*Sat15*^ (L) or *fru*^*ΔC*^*/fru*^*Sat15*^ (M) gonads showing residual FruCom staining in these alleles. (N-O) Late L3 stage *fru* null (*fru*^*Sat15*^*/fru*^*W24*^) male (N) and female (O) gonads showing the specificity of the anti-Fru antibody. (P) RT-PCR of adult testes with promoter-specific primers. *fruCOM* primers were used as positive controls. Faint bands for P1 and P2 are likely due to contamination of neurons innervating the testes. Scale bars represent 20 μm. Circle denotes the hub; brackets denote the TF.
(TIF)

S2 Fig Immunostaining as indicated in the figure and described previously. Msl2-GFP (blue) is used to determine sex and is part of the X chromosome dosage compensation complex that labels the X chromosome in males which can be observed as a nuclear focus of fluorescence that is distinct from Vasa (same channel). (A) A representative XY *dsx* heterozygote GSC niche showing wild-type level of Fru expression. (B) A representative *XX; Df(3R)dsx*^*3*^*/dsx*^*1*^ gonad with the male niche identity showing Fru expression in the hub cells and early CySC lineage at a reduced level. (C-D) representative images showing *XY; Df(3R)dsx*^*3*^*/dsx*^*1*^ gonads with a hub have variable Fru expression levels. All images represent late L3 stage gonads. Scale bars represent 20 μm. Circle denotes the hub; brackets denote the TF; arrows denote CySCs.
(TIF)

S3 Fig (A-B) White prepupal stage *fru* het (A) and *fru* null (B) testes. (C-D) Representative images of *fru* het (C) and *fru*^*ΔB*^*/fru*^*Sat15*^ mutant (D) testes 3 days after puparium formation. (E-G) 1-week old testis with *UAS-fruCom RNAi* (E) alone, or expressing *fruCom RNAi* in the hub with *upd-Gal4* (F) or expressing *fruCom RNAi* in the hub and early CySC lineage with *tj-Gal4* (G). (H-I) 2-week old testes expressing *GFP RNAi* (H) or *fruCom RNAi* (I) with *tj-Gal4*. (J) Quantification of the length of Tj+ zone in control and *fruCom RNAi* testes. Mean ± SD, Student’s t-test. Scale bars represent 20 μm. Circle denotes the hub.
(TIF)

S4 Fig (A) The *fru* promoter region is shown to scale with transcripts generated from *P1-P4* are labeled. (B) Putative Dsx binding motifs shown as top 1% position weight matrix (PWM), top 10% PWM and evolutionarily conserved Dsx motifs [15]. The three potential Dsx binding sites that were mutated were squared in red. Dsx direct binding in the fru locus was indicated by (C) female and male fate body Dsx-DamID and (D) S2 cells Dsx^M^ and Dsx^F^ ChIP-Seq peaks.
(TIF)

S5 Fig (A, B) Immunostaining of *dsx-*Gal4 crossed to UAS-GFP.nls to indicate the *dsx-Gal4* expression pattern in male and female L3 larval gonads. (C-F) Immunostainings of L3 larval ovaries to control for expression levels of FRU isoforms. The anti-FruCOM antibody was used for all. *tj-Gal4* control (C) and different isoforms of FRU driven by *tj-Gal4* as indicated (D-F).
(TIF)

S6 Fig (A) Evolutionary conservation analyses of DSX1 using comparative genomics tracks of the UCSC Genome Browser. Sequence alignment among *Drosophila* species is shown with same nucleotides abbreviated as dots. (B-D) GFP expression of *P4* WT (B), Mut1 (C) and Mu123 (D) constructs in late L3 stage ovaries. Scale bars represent 20 μm. Brackets denote TFs. (E) Comparison of relative GFP fluorescent intensity per hub cells (standardized by Piwi expression) in WT, Mut1 and Mut 123 constructs (as done in Fig 6). Bars represent Mean±SEM. Sample size: WT, n = 50; Mut1, n = 35; Mut123, n = 40. Student’s t-test.
(TIF)

S1 Table Quantification of niche sex identity in *XX*; *dsx*^*D*^*/+* and *XY; Df(3R)dsx*^*3*^*/dsx*^*1*^ adult gonads. (TIF)

S2 Table Quantification of control and *fru* clones. (TIF)
