# Supplementary figures and images for: Doublesex regulates fruitless expression to promote sexual dimorphism of the gonad stem cell niche
Source: PLoS Genet. 2021 Mar 31;17(3):e1009468. doi: 10.1371/journal.pgen.1009468 (PMC8041189; doi:10.1371/journal.pgen.1009468)

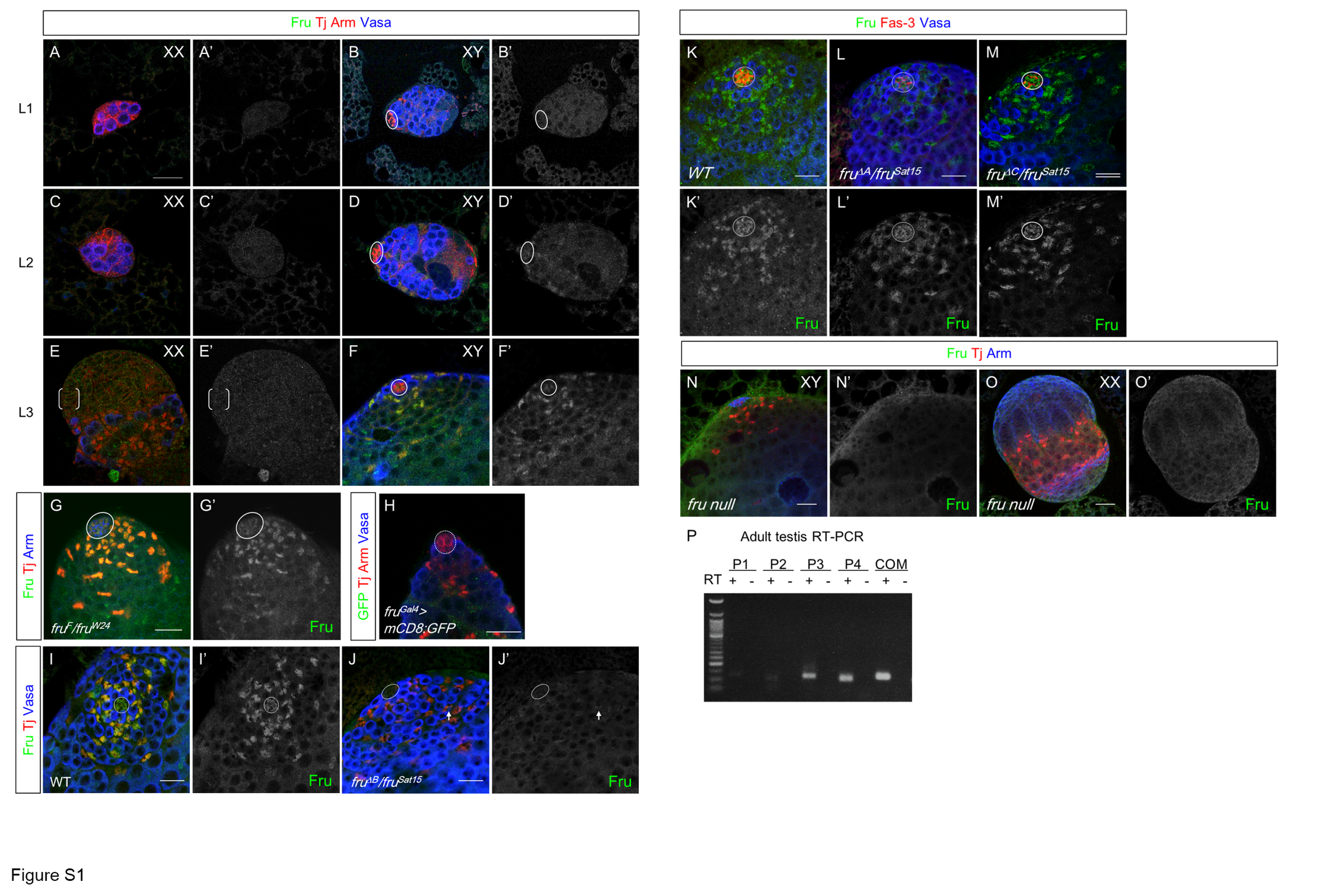

Supplement: S1 Fig — Anti-Vasa labels the germline, Anti-Arm labels the hub, anti-Tj labels the CySC and early cyst cells of the testes along with the somatic cells intermingled with germ cells in the ovary. (A-B) Wildtype L1 stage female and male gonads with no Fru expression. (C-D) Wildtype L2 stage gonads showing weak Fru expression in the hub cells and early CySC lineage of the testis. (E-F) Wildtype late L3 stage gonads showing robust Fru expression in the male GSC niche and no Fru expression in the female GSC niche (G) A representative fruF/fruW24 adult testis showing normal Fru expression in the niche. (H)A representative fruGal4>mCD8:GFP testis showing no GFP expression in the niche. (I-J) Late L3 stage wildtype (I) and fruΔB/fruSat15 (J) gonads showing the reduced FruCom immunoreactivity in the hub and the Tj+ cyst cells (arrow). (K-M) Late L3 stage wildtype (K) and fruΔA/fruSat15 (L) or fruΔC/fruSat15 (M) gonads showing residual FruCom staining in these alleles. (N-O) Late L3 stage fru null (fruSat15/fruW24) male (N) and female (O) gonads showing the specificity of the anti-Fru antibody. (P) RT-PCR of adult testes with promoter-specific primers. fruCOM primers were used as positive controls. Faint bands for P1 and P2 are likely due to contamination of neurons innervating the testes. Scale bars represent 20 μm. Circle denotes the hub; brackets denote the TF. (TIF) [file pgen.1009468.s001.tif]

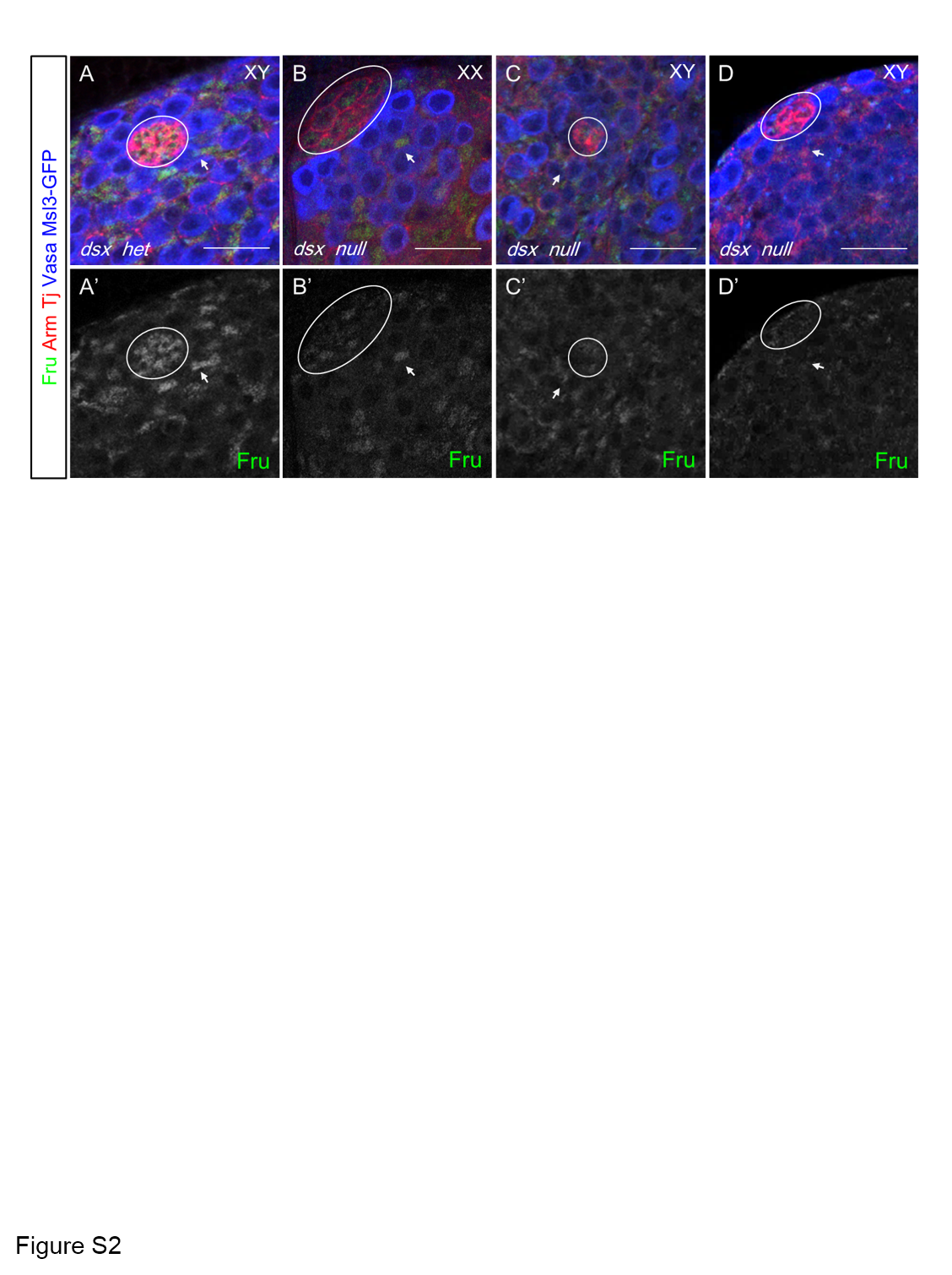

Supplement: S2 Fig — Msl2-GFP (blue) is used to determine sex and is part of the X chromosome dosage compensation complex that labels the X chromosome in males which can be observed as a nuclear focus of fluorescence that is distinct from Vasa (same channel). (A) A representative XY dsx heterozygote GSC niche showing wild-type level of Fru expression. (B) A representative XX; Df(3R)dsx3/dsx1 gonad with the male niche identity showing Fru expression in the hub cells and early CySC lineage at a reduced level. (C-D) representative images showing XY; Df(3R)dsx3/dsx1 gonads with a hub have variable Fru expression levels. All images represent late L3 stage gonads. Scale bars represent 20 μm. Circle denotes the hub; brackets denote the TF; arrows denote CySCs. (TIF) [file pgen.1009468.s002.tif]

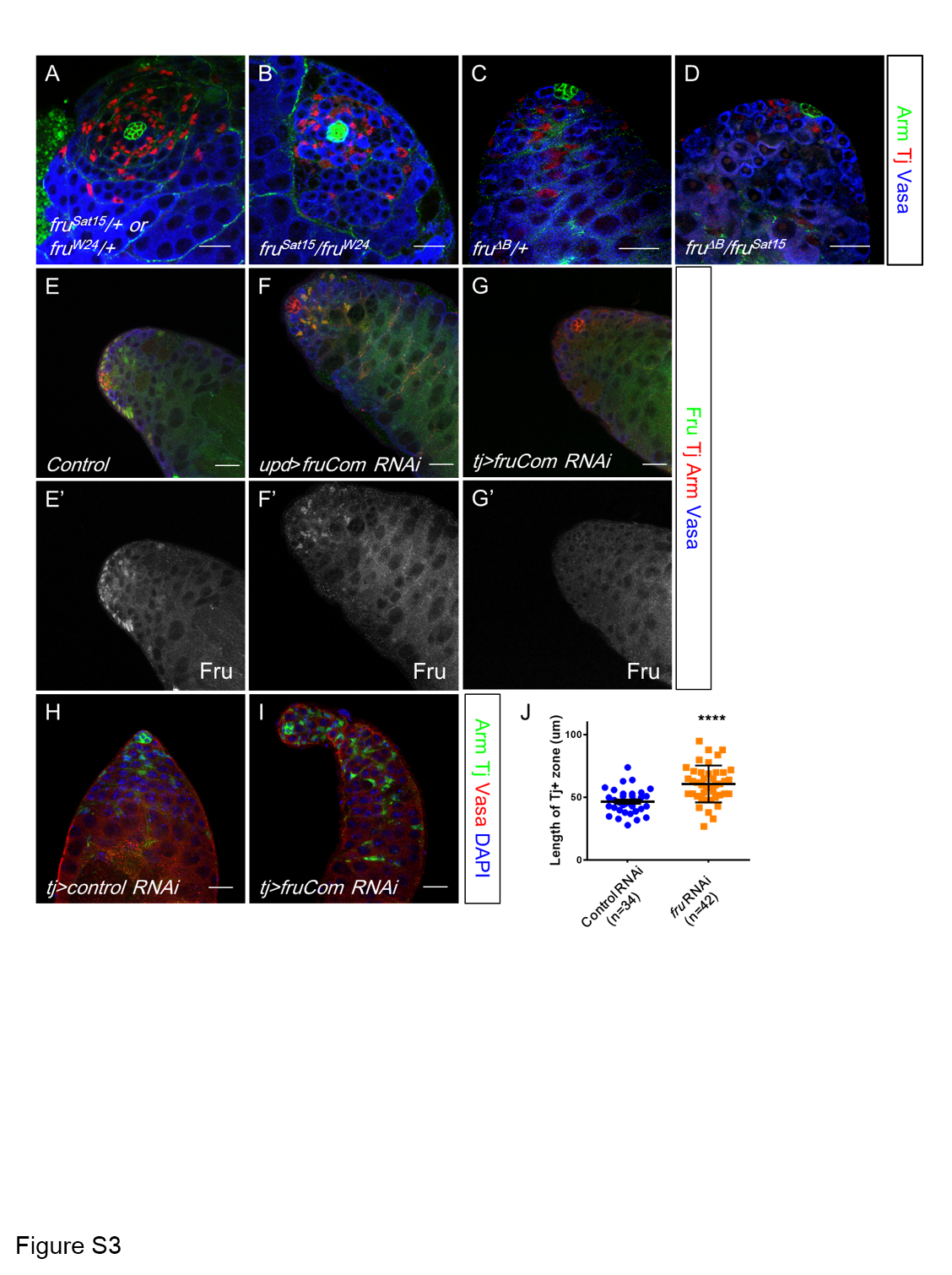

Supplement: S3 Fig — (A-B) White prepupal stage fru het (A) and fru null (B) testes. (C-D) Representative images of fru het (C) and fruΔB/fruSat15 mutant (D) testes 3 days after puparium formation. (E-G) 1-week old testis with UAS-fruCom RNAi (E) alone, or expressing fruCom RNAi in the hub with upd-Gal4 (F) or expressing fruCom RNAi in the hub and early CySC lineage with tj-Gal4 (G). (H-I) 2-week old testes expressing GFP RNAi (H) or fruCom RNAi (I) with tj-Gal4. (J) Quantification of the length of Tj+ zone in control and fruCom RNAi testes. Mean ± SD, Student’s t-test. Scale bars represent 20 μm. Circle denotes the hub. (TIF) [file pgen.1009468.s003.tif]

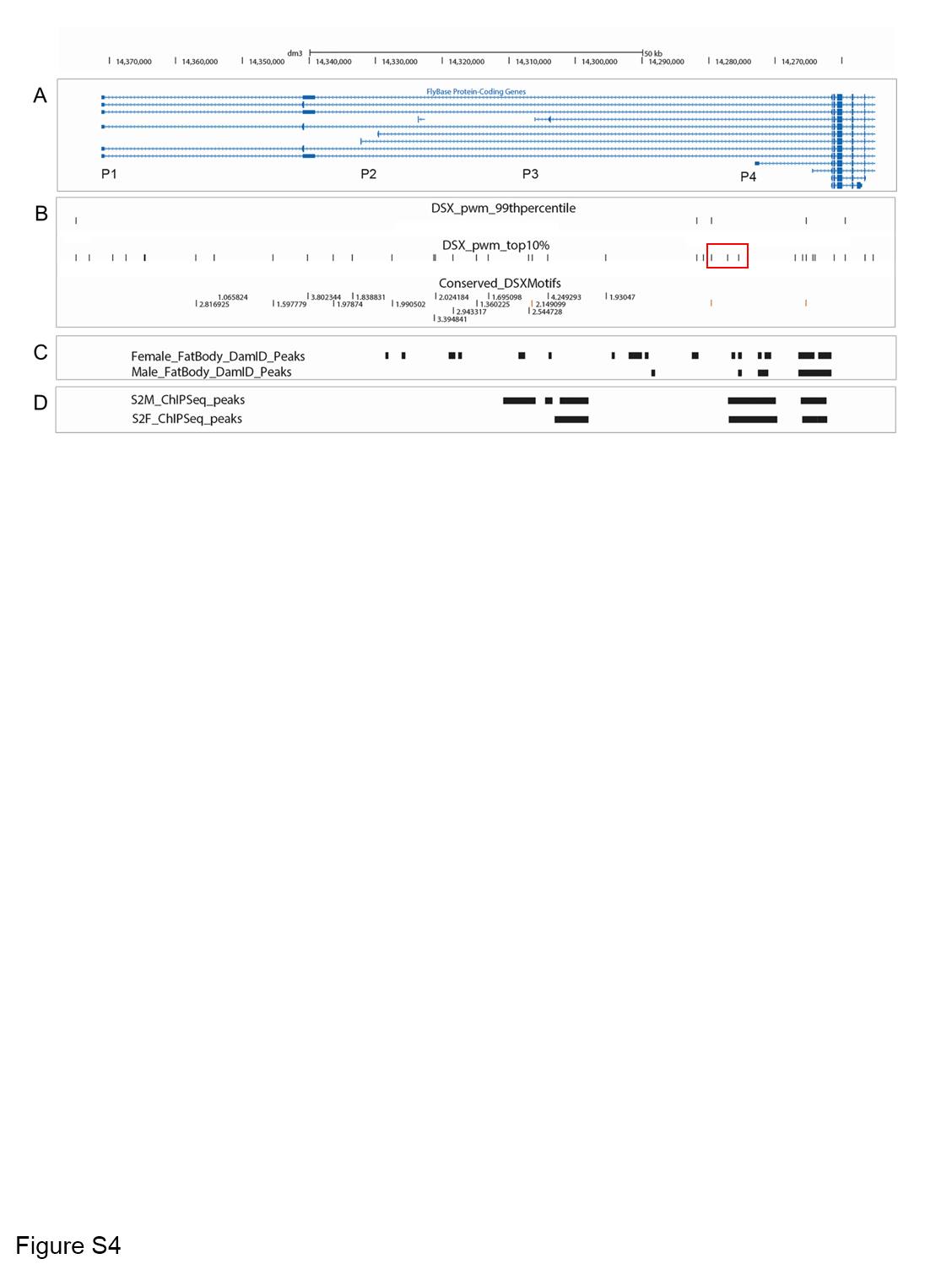

Supplement: S4 Fig — (A) The fru promoter region is shown to scale with transcripts generated from P1-P4 are labeled. (B) Putative Dsx binding motifs shown as top 1% position weight matrix (PWM), top 10% PWM and evolutionarily conserved Dsx motifs [15]. The three potential Dsx binding sites that were mutated were squared in red. Dsx direct binding in the fru locus was indicated by (C) female and male fate body Dsx-DamID and (D) S2 cells DsxM and DsxF ChIP-Seq peaks. (TIF) [file pgen.1009468.s004.tif]

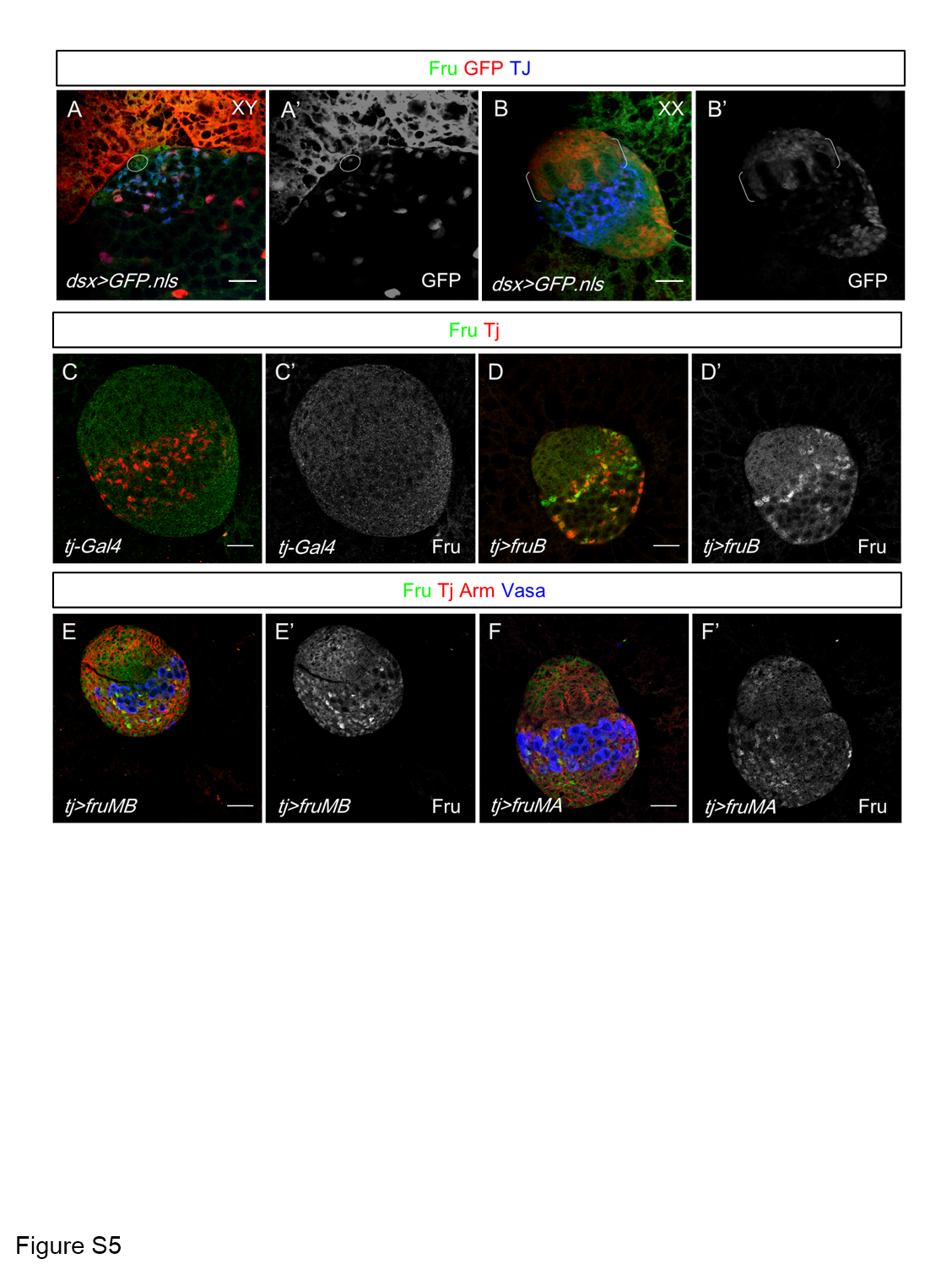

Supplement: S5 Fig — (A, B) Immunostaining of dsx-Gal4 crossed to UAS-GFP.nls to indicate the dsx-Gal4 expression pattern in male and female L3 larval gonads. (C-F) Immunostainings of L3 larval ovaries to control for expression levels of FRU isoforms. The anti-FruCOM antibody was used for all. tj-Gal4 control (C) and different isoforms of FRU driven by tj-Gal4 as indicated (D-F). (TIF) [file pgen.1009468.s005.tif]

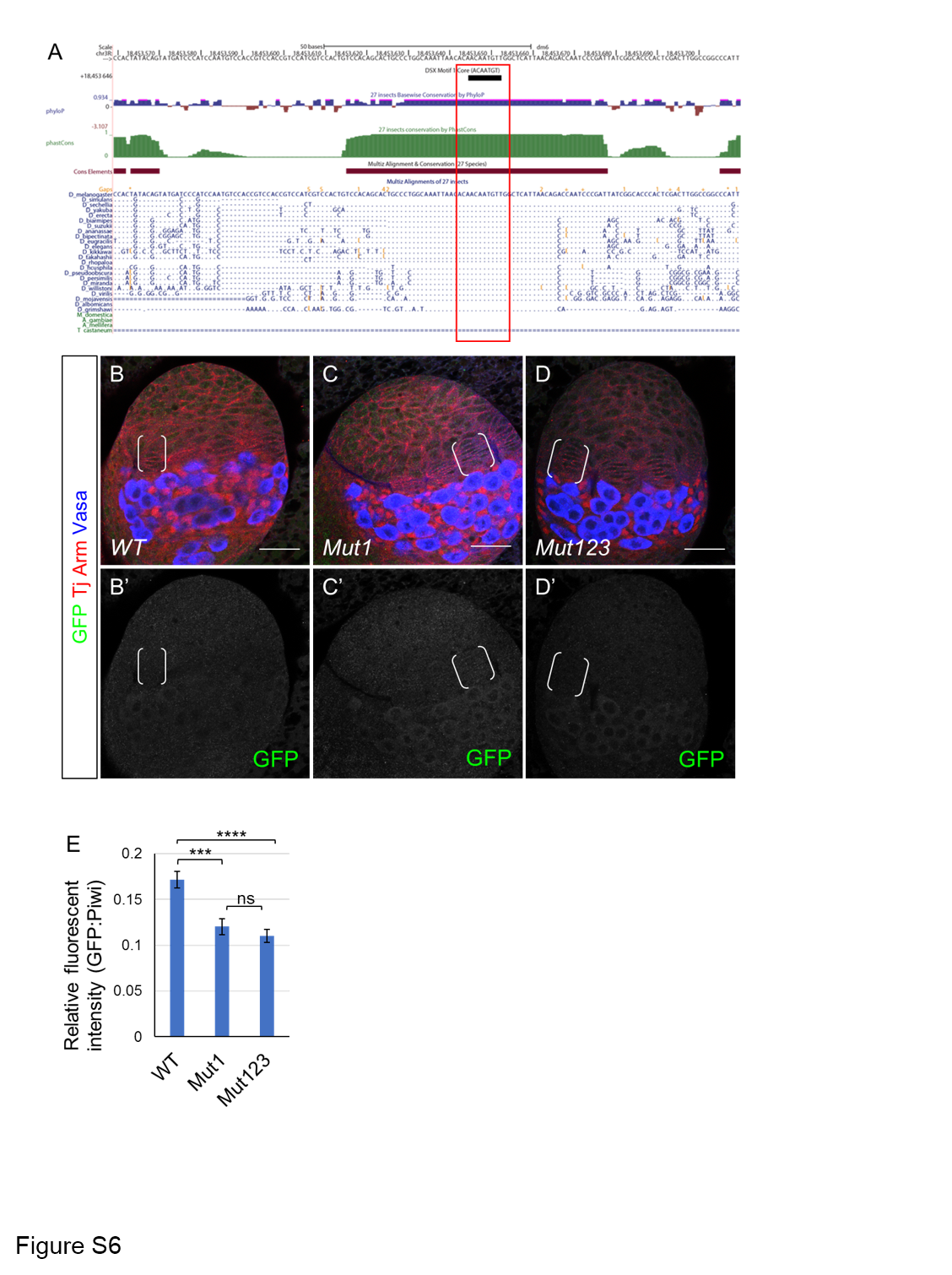

Supplement: S6 Fig — (A) Evolutionary conservation analyses of DSX1 using comparative genomics tracks of the UCSC Genome Browser. Sequence alignment among Drosophila species is shown with same nucleotides abbreviated as dots. (B-D) GFP expression of P4 WT (B), Mut1 (C) and Mu123 (D) constructs in late L3 stage ovaries. Scale bars represent 20 μm. Brackets denote TFs. (E) Comparison of relative GFP fluorescent intensity per hub cells (standardized by Piwi expression) in WT, Mut1 and Mut 123 constructs (as done in Fig 6). Bars represent Mean±SEM. Sample size: WT, n = 50; Mut1, n = 35; Mut123, n = 40. Student’s t-test. (TIF) [file pgen.1009468.s006.tif]

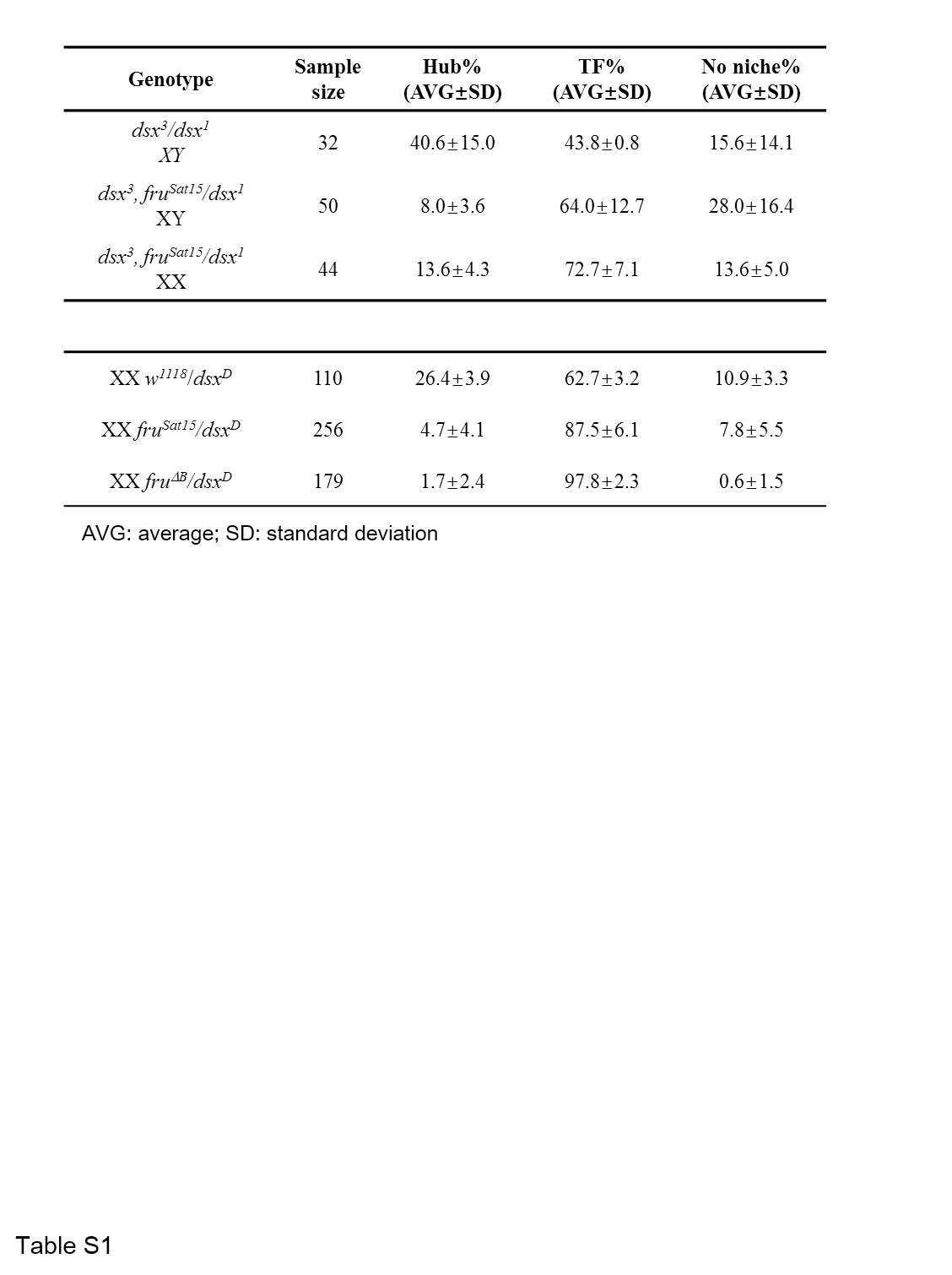

Supplement: S1 Table — (TIF) [file pgen.1009468.s007.tif]

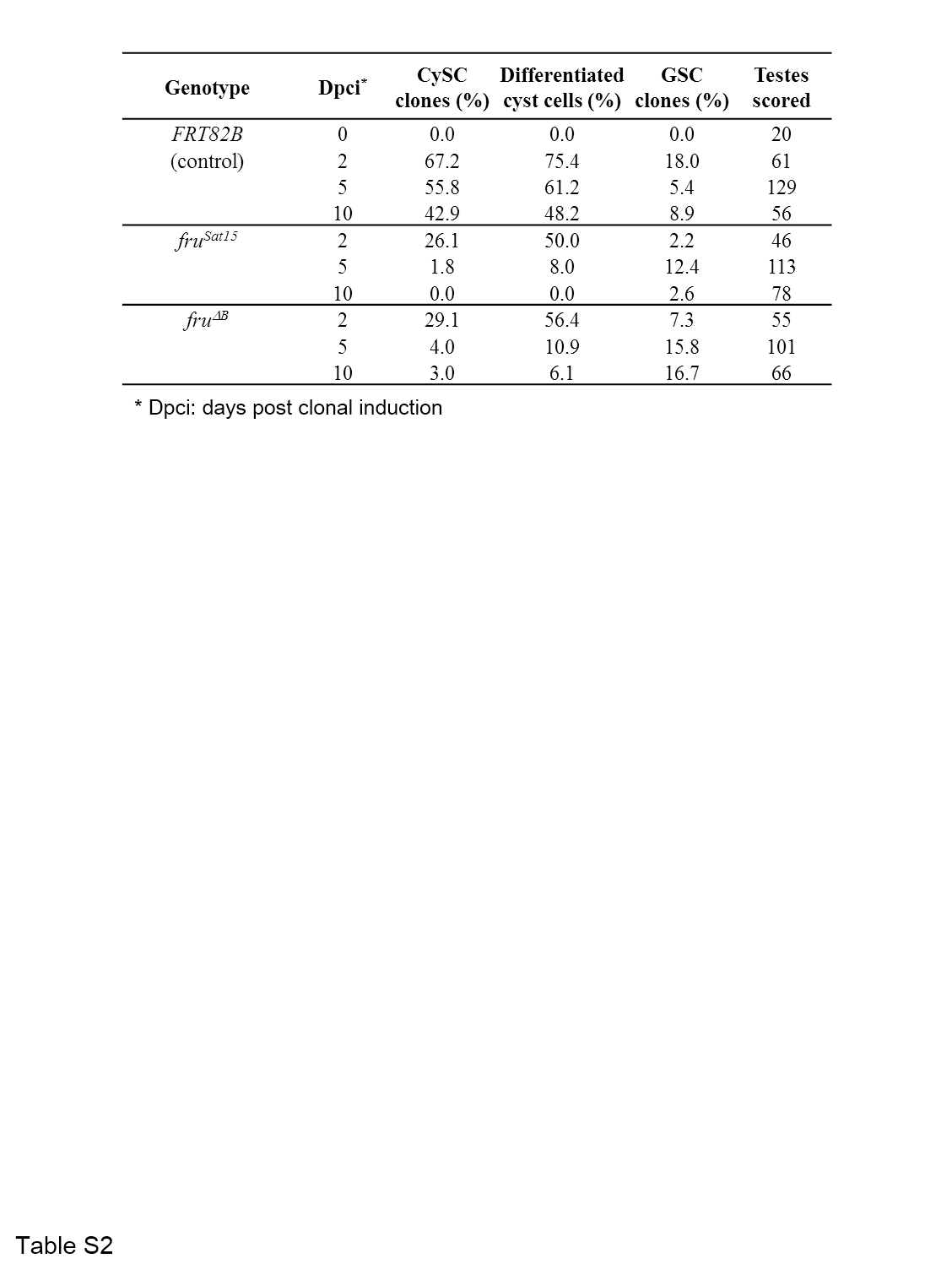

Supplement: S2 Table — (TIF) [file pgen.1009468.s008.tif]
